# Interventions to improve continence for children and young people with neurodisability: a national survey of practitioner and family perspectives and experiences

**DOI:** 10.1136/bmjpo-2023-002238

**Published:** 2024-01-19

**Authors:** Helen Eke, Susan Ball, Annette Allinson, Rob Anderson, Harriet Hunt, Eve Hutton, Claire Lindsay, Stuart Logan, Nicholas Madden, Julia Melluish, Davina Richardson, June Rogers, Jo Thompson Coon, Rebecca Whear, Anne Wright, Christopher Morris

**Affiliations:** 1 Medical School, University of Exeter, Exeter, UK; 2 National Institute for Health Research (NIHR) Applied Research Collaboration (ARC) South West Peninsula (PenARC), University of Exeter, Exeter, UK; 3 Peninsula Childhood Disability Research Unit, University of Exeter, Exeter, UK; 4 Canterbury Christ Church University, Canterbury, UK; 5 Northern Devon Healthcare NHS Trust, Exeter, UK; 6 Chelsea and Westminster Hospital NHS Foundation Trust, London, UK; 7 Disabled Living Foundation, London, UK; 8 Guy's and St Thomas' Hospitals NHS Trust, London, UK

**Keywords:** Neurodisability, Enuresis, Health services research

## Abstract

**Objective:**

Describe families’ experiences of interventions to improve continence in children and young people with neurodisability, and health professionals’ and school and social care staff’s perspectives regarding factors affecting intervention use.

**Design:**

Four online surveys were developed and advertised to parent carers, young people with neurodisability, health professionals and school and social care staff, via societies, charities, professional contacts, schools, local authorities, and national parent carer and family forums, who shared invitations with their networks. Survey questions explored: difficulties helping children and young people use interventions; acceptability of interventions and waiting times; ease of use and availability of interventions, and facilitators and barriers to improving continence.

**Results:**

1028 parent carers, 26 young people, 352 health professionals and 202 school and social care staff registered to participate. Completed surveys were received from 579 (56.3%) parent carers, 20 (77%) young people, 193 (54.8%) health professionals, and 119 (58.9%) school and social care staff. Common parent carer-reported difficulties in using interventions to help their children and young people to learn to use the toilet included their child’s lack of understanding about what was required (reported by 337 of 556 (60.6%) parent carers who completed question) and their child’s lack of willingness (343 of 556, 61.7%). Almost all (142 of 156, 91%) health professionals reported lack of funding and resources as barriers to provision of continence services. Many young people (14 of 19, 74%) were unhappy using toilet facilities while out and about.

**Conclusions:**

Perceptions that children lack understanding and willingness, and inadequate facilities impact the implementation of toileting interventions for children and young people with neurodisability. Greater understanding is needed for children to learn developmentally appropriate toileting skills. Further research is recommended around availability and acceptability of interventions to ensure quality of life is unaffected.

**WHAT IS ALREADY KNOWN ON THIS TOPIC**

- Children with neurodisability are often delayed in acquiring continence and are more frequently incontinent than typically developing children. Social, economic and environmental factors, parenting strategies and child behaviour all affect toilet training.
- Incontinence can affect the quality of life of the child or young person and their carers.
- Many children with neurodisability can become continent, and while not all children are able to become fully independent in toileting, many can improve their continence with support, with consequent positive gains in quality of life.
- A variety of approaches and interventions are available including information and support, charts to monitor/feedback, schedules for drinking and toileting, cognitive–behavioural approaches, equipment, aids, relaxation techniques, psychotherapy, group-based programmes, medicines and surgery. Previous research has identified limited evidence for toilet training strategies for children with physical and learning disabilities.

**WHAT THIS STUDY ADDS**

- Families describe inadequate access to continence support and interventions.
- Many children are perceived as lacking willingness and understanding regarding continence needs.
- Many health professionals reported poor local availability of some interventions and a lack of funding for dedicated continence services.
- Children and young people were unhappy with some interventions and with out-of-home toilet facilities.

**HOW THIS STUDY MIGHT AFFECT RESEARCH, PRACTICE OR POLICY**

- Highlights the need for further research around acceptability of interventions to improve continence in children and young people with neurodisability, without compromising quality of life.
- Emphasises the requirement for an individual assessment of needs for children and young people with neurodisability, to be carried out proactively and in a timely manner.
- Funding issues are likely restricting the toileting interventions that are being offered to children and young people with neurodisability.

## Introduction

Neurodisability describes a group of congenital or acquired long-term conditions attributed to the impairment of the brain and/or neuromuscular system, creating functional limitations including difficulties with movement, cognition, hearing, vision, communication, emotion and behaviour.^1^ Children with neurodisability are often delayed in acquiring continence, and more frequently incontinent than typically developing children; they may be slower to learn to manage toileting or may need additional support.^1–3^ Social, economic and environmental factors, parenting strategies and child behaviour can all affect toilet training.

Continence is being clean and dry without using containment products such as pads/nappies. Incontinence can affect the quality of life of the young person and their carers,^4^ and the long-term physical, psychological and financial burden can be considerable.^5^ The spectrum of neurodisability is vast and some children may never reach a physical or psychological developmental stage where continence is an achievable goal. Many children with neurodisability however can become continent, and while not all are able to become fully independent in toileting, many can improve their continence with support,^2–4^ with consequent improvement in quality of life. Distinguishing continence for individuals with and without spinal cord pathology affecting bladder and bowel sensorimotor control is therefore crucial. Without sensation and motor control, and normal detrusor, colonic and sphincter function, assistive technology or alternative approaches to bladder and bowel storage and emptying will often be needed.

A variety of approaches and interventions are available^6^ including information and support, monitoring/feedback charts, drinking and toileting schedules, cognitive–behavioural approaches, equipment, aids, relaxation techniques, psychotherapy, group-based programmes, medicines and surgery.^7^ An existing systematic review identified limited evidence for toilet training strategies for children with physical and learning disabilities.^8^

Research to evaluate ways to promote continence for children with neurodisability ranked 7 out of 10 in the British Academy of Childhood Disability James Lind Alliance Research Priority Setting Partnership.^9^ Subsequently, the ICoN (Improving Continence in children and young people with Neurodisability) Study was commissioned by the National Institute for Health and Care Research to summarise available evidence for interventions and practice relating to improving continence for children and young people with neurodisability.^10^ Online surveys were conducted with parent carers, children and young people with neurodisability, health professionals (HP) and school and social care staff (SSC). This paper details the survey process and aims to describe the experiences and perspectives of parent carers and children and young people with neurodisability regarding interventions to improve continence, and to explore health professionals’ and SSC’s perspectives regarding factors affecting intervention use.

## Methods

### Survey development

Survey questions and response options were developed in collaboration with the ICoN Family Faculty public involvement group, Professional Advisory Group and young people with neurodisability. Surveys were developed through iteration and piloting.

The surveys included adaptive questioning; respondents were only asked to answer questions that were relevant to them. Parent carers, HPs and SSC completed an initial eligibility question (related to their experience of continence support and neurodisability), and chose a clinical group about which to complete the survey; non-spinal cord pathology, defined as *social/communication or attention/behaviour difficulty, learning disability or physical or movement disability*, or spinal cord pathology, defined as *bladder and/or bowel impairment due to damage to the spinal cord, for example, neurogenic/neuropathic condition*. HPs had the option to complete the survey for both clinical groups.

Question order was fixed within surveys, following a logical order in terms of the respondent characteristics and experiences. Surveys included a maximum of five demographic characteristics questions, and up to 14 questions about professional practice and/or experience regarding interventions for improving continence. The children and young people’s survey included just four questions regarding toileting abilities and experiences. The HP and SSC surveys included 16 additional questions regarding service provision, which all respondents, regardless of chosen clinical group, were asked to complete. HP survey questions reported in this paper come from the service provision section, not separated by clinical group. Box 1 lists the survey questions covered in this paper.

Box 1 **List of survey questions included in analyses**

**Survey questions included in this paper**

*Parent carers*

- What difficulties have you found using methods to help with toileting at home?
- If you and your child have experienced any of the following methods to help with toileting, please indicate how easy you found it using them at home.
- Where you live, please indicate which toileting products or equipment are provided for families of children and young people with special educational needs and/or a disability.
- Where you live, how acceptable is the waiting time for families to get the equipment and products they require once an assessment has been completed?

*Children and young people*

- How do you feel about (list of different interventions)?
- How do you feel about using the toilet (list of different environments)?

*Health professionals*

- Where you work, please indicate which toileting products or equipment are provided for families of children and young people with special educational needs and/or a disability.
- In your opinion, how acceptable is the waiting time in your area for families to receive the toileting support or equipment/products they require?
- In your opinion, to what extent do local commissioning/funding arrangements influence the toileting support you offer for individual children and young people with special educational needs and/or a disability (eg, would you recommend the use of continence pads as they are supplied free of charge for families locally)?
- Where you work, do you have a bladder and bowel protocol/pathway for children and young people with special educational needs and/or a disability?
- Where you work, are there any toileting interventions for children and young people with special educational needs and/or a disability that are not provided, which you know are provided elsewhere?
- In your opinion, what do you think is the main barrier to developing a dedicated bladder and bowel pathway or service?
- In your opinion, is the provision of continence pads for children and young people with special educational needs and/or a disability a barrier or enabler for achieving continence?

*School and social care staff*

- Where you work, what difficulties have you found in helping children and young people to use the toileting methods (eg, alarms or frames) provided?
- Where you work, how easy is it for you to provide or use the following methods to help children and young people with toileting?
- Where you work, please indicate which toileting products or equipment are provided for families of children and young people with special educational needs and/or a disability.

### Participants and procedure

Parent carers, children and young people, HPs and SSC were invited to participate via a study advert, shared by over 100 societies, charities, organisations, professional contacts, schools, local authorities and national parent carer and family forums. Study information was also advertised regularly by the research team, via social media. A copy of the study advert can be found in the online supplemental material. This targeted recruitment gathered a diverse sample of participants with knowledge and experience of continence services and interventions for children and young people with neurodisability.

10.1136/bmjpo-2023-002238.supp1 Supplementary data

Participants registered via a study website (https://blogs.exeter.ac.uk/iconstudy/) to be emailed a personalised link to complete the relevant survey. This registration process created a ‘closed’ survey, allowing the study team to know to whom the invitations were sent, rather than an open survey for anyone to answer. Monitoring of registered email addresses allowed identification of duplicate registrations, and up to four reminders were sent to non-responders. Each participant was assigned a unique identifier via the registration website, preventing participants from completing the survey more than once. No analysis of IP addresses or log files was conducted.

Informed consent was assumed through voluntary registration online.^11^ Participants were encouraged to read the participant information sheet available via the registration web page. Participation was voluntary, and no participation incentive was offered. The time required to complete the survey was not recorded although participants were advised that the survey required mostly tick box responses and would take approximately 15 minutes. Participants were able to review and save their responses prior to submission. All individually identifiable data were password protected via the registration website.

### Data collection and reporting

The surveys were open between July and December 2019.

Numbers of registrations and completed surveys were recorded. Data were exported into Microsoft Excel and analysed using R.^12^ Guidelines for reporting online surveys were followed^13^ (see online supplemental material for a copy of the completed Checklist for Reporting Results of Internet E-Surveys).

10.1136/bmjpo-2023-002238.supp2 Supplementary data

The number and percentage of completed surveys (as a proportion of personalised links sent) are reported. Demographic characteristics are summarised and the number of responses to each survey question reported. As not all questions were mandatory, the total number of responses varies across questions. Responses of ‘don’t know’, ‘never used’, ‘no knowledge’ or ‘never happened to me’ regarding a service or intervention are excluded from the description of results but are included in results tables. Parent carer survey responses are reported by clinical group. HP survey responses are reported by job role. Selected free-text responses illustrate salient results from the numerical data.

## Results

### Survey responses

In total, 605 responses were received from 1028 parent carer registrations, 20 from 26 registered children and young people, 202 from 352 HP registrations and 122 from 202 SSC registrations. Nine responses were excluded from both the parent carer and HP survey and three from the SSC survey due to ineligibility (no experience of continence support for, or the care of, children and young people with neurodisability). A further 17 parent carer responses were excluded because their child did not belong to one of the two clinical groups (n=10) or could not be allocated to one of these groups from their free-text description of their child’s condition (n=7). Completed survey responses are therefore reported from 579 (56.3%) parent carers, 20 (77%) children and young people, 193 (54.8%) HPs and 119 (58.9%) SSC.

### Characteristics of respondents

Ninety percent (522 of 579) of parent carers were the child’s mother, 396 (68.4%) of the children about whom the parent carers were completing the survey were male, and 446 (77%) were aged between five and 17 years. Over two-thirds of children and young people respondents were female (14 of 20), of white ethnic origin (19 of 20), aged over 18 years (14 of 20) and were identified as being in the non-spinal cord pathology clinical group (17 of 20) from a free-text description of their condition. HPs were grouped into six categories by job role: bladder and bowel specialist (BBS) nurse, nurse, paediatrician, surgeon, therapist, other. Details of the roles included within these categories are provided elsewhere.^9^ Common SSC roles were teachers (29 of 119, 24.4%), assistants (28 of 119, 23.5%) and ‘others’ (36 of 119, 30.3%), and almost half worked in special schools (51 of 119, 42.9%). All geographical regions of England were represented (table 1).

**Table 1:** Characteristics of survey respondents

Parent carers		Children and young people		Health professionals		School and social care staff	
**Characteristic**	**N=579**	**Characteristic**	**N=20**	**Characteristic**	**N=193**	**Characteristic**	**N=119**
**Child’s gender**		**Gender**		**Gender**		**Gender**	
Male	396 (68.4)	Male	6 (30)	Male	16 (8.3)	Male	6 (5.0)
Female	181 (31.3)	Female	14 (70)	Female	158 (81.9)	Female	111 (93.3)
pnts	2 (0.3)	pnts	0 (0)	pnts	7 (3.6)	pnts	1 (0.8)
na	0 (0)	na	0 (0)	na	12 (6.2)	na	1 (0.8)
**Region of England where live**		**Region of England where live**		**Region of England where work**		**Region of England where work**	
North West	40 (6.9)	North West	3 (15)	North West	21 (10.9)	North West	10 (8.4)
North East	21 (3.6)	North East	1(5)	North East	12 (6.2)	North East	4 (3.4)
Y & H	31 (5.4)	Y & H	2 (10)	Y & H	9 (4.7)	Y & H	5 (4.2)
East Midlands	32 (5.5)	East Midlands	0 (0)	East Midlands	9 (4.7)	East Midlands	2 (1.7)
West Midlands	53 (9.2)	West Midlands	0 (0)	West Midlands	7 (3.6)	West Midlands	3 (2.5)
East Anglia	26 (4.5)	East Anglia	1 (5)	East Anglia	9 (4.7)	East Anglia	2 (1.7)
South West	166 (28.7)	South West	4 (20)	South West	39 (20.2)	South West	60 (50.4)
South East	123 (21.2)	South East	4 (20)	South East	26 (13.5)	South East	22 (18.5)
London	48 (8.3)	London	1 (5)	London	38 (19.7)	London	6 (5.0)
Outside England	28 (4.8)	Outside England	4 (20)	Outside England	18 (9.3)	Outside England	2 (1.7)
na	11 (1.9)	na	0 (0)	na	5 (2.6)	na	3 (2.5)
**Age of child (years)**		**Age (years)**				**Job role**	
Under 5	68 (11.7)	Under 5	0 (0)			Manager	19 (16.0)
5–7	136 (23.5)	5–7	0 (0)			Assistant	28 (23.5)
8–11	153 (26.4)	8–11	1 (5)			Teacher	29 (24.4)
12–17	157 (27.1)	12–17	5 (25)			Enabler	5 (4.2)
18–25	64 (11.1)	18–25	11 (55)			Social worker	0 (0)
Over 25	0 (0.0)	Over 25	3 (15)			Therapist	1 (0.8)
na	1 (0.2)	na	0 (0)			Other	36 (30.3)
**Ethnicity**		**Ethnicity**				na	1 (0.8)
White	540 (93.3)	White	19 (95)			**Where work**	
Mixed	13 (2.2)	Mixed	0 (0)			Mainstream school	30 (25.2)
Asian	10 (1.7)	Asian	0 (0)			Special school	51 (42.9)
Black	8 (1.4)	Black	0 (0)			Hospice/respite care	0 (0)
Other	6 (1.0)	Other	1 (5)			Short break facility	5 (4.2)
na	2 (0.3)	na	0 (0)			Community care	6 (5.0)
**Relationship to child**		**How completed survey**				Primary care	1 (0.8)
Mother*	522 (90.2)	On my own	17 (85)			Secondary care	0 (0)
Father	25 (4.3)	Someone is helping me	2 (10)			Other	25 (21.0)
Parent^†^	15 (2.6)	Someone is doing it for me	1 (5)			na	1 (0.8)
Grandparent	10 (1.7)	na	0 (0)				
Carer	2 (0.3)						
na	5 (0.9)						

Values are n (%) in respondent group.

*Including foster, adoptive and stepmother.

†Including foster parent.

Y & H, Yorkshire & Humberside; na, not answered; pnts, prefer not to say.

### Difficulties in helping children and young people use interventions: parent carers’ and SSC’s perspectives

The pattern of responses from parent carers regarding difficulties in helping children and young people to use toileting methods was similar for both clinical groups. The most common difficulties reported were the child’s lack of understanding (337 of 556, 60.6%) and willingness (343 of 556, 61.7%). Regarding services, the most frequently reported difficulties were access to appropriate help (240 of 556, 43.2%); lack of consistency of support in different environments (209 of 556, 37.6%); delays in professional assessments (151 of 556, 27.2%); and lack of funding for equipment and products (129 of 556, 23.2%). The most common SSC-reported difficulties included children and young people’s lack of understanding of what was required (67 of 115, 58.3%), limited parent carer capability and time (70 of 115, 60.9%), and lack of appropriate facilities at school (69 of 115, 60%) (table 2).

**Table 2:** Parent carers’ and school and social care staff’s perspectives on what difficulties they have found in helping children and young people to use toileting methods

Response option, n (% of respondents)	Parent carers		School and social care staff
	Non-spinal cord pathology	Spinal cord pathology	
	**N=536**	**N=20**	**N=115**
No difficulties experienced	34 (6)	4 (20)	16 (14)
Child/young person’s knowledge and understanding of what is required	328 (61)	9 (45)	67 (58)
Parent carer knowledge and understanding of what is required	56 (10)	4 (20)	54 (47)
Parent carer ability and time to focus on toileting	164 (31)	6 (30)	70 (61)
Child/young person’s willingness*	329 (61)	14 (70)	–
Child/young person’s adherence to the intervention^†^	–	–	48 (42)
Parent carer and child’s lack of interest/motivation to change	67 (13)	1 (5)	40 (35)
Not enough training in how to use the methods offered*	52 (10)	1 (5)	–
Delays in professional assessments	149 (28)	2 (10)	44 (38)
Access to appropriate help and support	233 (43)	7 (35)	38 (33)
Funding and/or resources for equipment and products	126 (24)	3 (15)	53 (46)
Lack of consistency in support in different environments, for example, facility at home but not at school	205 (38)	4 (20)	69 (60)
Other	47 (9)	1 (5)	5 (4)

Respondents were asked to choose all response options that applied.

*Response option included in parent carer survey only.

†Response option included in school and social care staff survey only.

### Acceptability of interventions: children’s and young people’s perspectives

Most children and young people with experience of toileting interventions felt happy or OK about using a hoist or frame (8 of 10), an alarm or timer (6 of 9), and half felt positively about using continence products and medications (6 of 12 for both). In contrast, many indicated unhappiness with following water/food diets (9 of 16), using a catheter or bowel washout (5 of 8) or having surgery (8 of 10). Most children and young people felt happy or OK using the toilet at home (14 of 18), but many felt negatively about using toilets at school, college or work (8 of 18), and out and about (14 of 19) (table 3).

**Table 3:** Children and young people’s perspectives on how they feel about toileting interventions and about using the toilet in different places

n (% of respondents)	Very happy	OK	A bit unhappy	Very unhappy	Don't know OR this has never happened to me
Intervention					
Following a special water/food diet (N=20)	2 (10)	5 (25)	7 (35)	2 (10)	4 (20)
Using an alarm or timer to remind me to wee/poo (N=20)	0 (0)	6 (30)	1 (5)	2 (10)	11 (55)
Using a hoist or a frame to help me use the toilet (N=20)	1 (5)	7 (35)	1 (5)	1 (5)	10 (50)
Using pads, nappies or pull ups (N=20)	3 (15)	3 (15)	1 (5)	5 (25)	8 (40)
Taking medication to help me wee/poo (N=20)	4 (20)	2 (10)	5 (25)	1 (5)	8 (40)
Using a catheter or bowel washout (tubes that help you to wee or poo) (N=20)	1 (5)	2 (10)	0 (0)	5 (25)	12 (60)
Having surgery (N=20)	2 (10)	0 (0)	2 (10)	6 (30)	10 (50)
Place					
Using the toilet at home (N=19)	12 (63)	2 (11)	3 (16)	1 (5)	1 (5)
Using the toilet at school/college/work (N=20)	5 (25)	5 (25)	3 (15)	5 (25)	2 (10)
Using the toilet when out and about, for example, in a restaurant (N=20)	2 (10)	3 (15)	9 (45)	5 (25)	1 (5)

Numbers are n (%) of respondents who gave an answer to the statement, so percentages add up to 100 across the rows.

### Ease of use of interventions: parent carers’ and SSC’s perspectives

In the non-spinal cord pathology group, parent carers reported that toileting products (320 of 401, 80%) and simple aids/equipment (216 of 292, 74%) were easiest to use. In the spinal cord pathology group, all five parent carers with experience of housing adaptations, and 15 of 16 with experience of continence products, reported them to be easy or very easy to use.

A large proportion of SSC with experience of interventions to promote continence found toileting products or training methods easy or very easy to use: dietary advice (76 of 96, 79%), fluid intake advice (88 of 106, 83%) and behavioural interventions (98 of 109, 89.9%). In contrast, only 31% of parent carers (93 of 299) found behavioural interventions easy or very easy to use (online supplemental table 1).

10.1136/bmjpo-2023-002238.supp3 Supplementary data

### Availability of interventions: parent carers’, SSC’s and HPs’ perspectives

Substantial numbers of HPs with knowledge of specific aids said they were not available or only available by purchase (behavioural aids such as alarms, 74 of 144 (51.4%); simple aids such as raised seats or steps, 71 of 146 (48.6%); and housing adaptations, 39 of 124 (31.4%)). The most common response regarding provision of continence products was that they were supplied free of charge (17 of 18, 94% BBS nurses; 11 of 17, 65% therapists; 55 of 93, 59% SSC; 256 of 374, 68.4% and 9 of 17, 53% parent carers for non-spinal cord and spinal cord pathologies, respectively) (online supplemental table 2).

### Acceptability of waiting times for continence support: parent carers’ and HPs’ perspectives

Roughly half of parent carers and HPs with knowledge of waiting times for continence support considered them acceptable (table 4). Parent carers and HPs who considered waiting times to be unacceptable described long waits for assessments, lack of funding and staffing:

*Waiting times are too long for assessment, and then provision of complex equipment and/or adaptations can also take a long time.* (Therapist 48880209)

*Not enough continence support so waiting times long and services are not funded*. (Nurse 49177881)

*Everything takes a very long time to come through, including assessment - not had one for years*. (Parent carer 49575343)

*Two part time staff for the whole county, very few appointments and long waiting list. Very restrictive criteria to access the service.* (Parent carer 50843591)

*Funding is always an issue. It’s always a fight to get appropriate services*. (Parent carer 50044306)

**Table 4:** Parent carers’ and health professionals’ perspectives on the acceptability of waiting times for families to receive the required toileting support or equipment/products

	Parent carers		Health professionals					
**Response option, n (% of respondent group**)	**Non-spinal cord pathology**	**Spinal cord pathology**	**BBS nurses**	**Nurses**	**Paediatricians**	**Surgeons**	**Therapists**	**Other**
	**N=553**	**N=20**	**N=22**	**N=79**	**N=30**	**N=15**	**N=33**	**N=13**
Very acceptable	27 (5)	3 (15)	4 (18)	5 (6)	0 (0)	2 (13)	2 (6)	0 (0)
Acceptable	121 (22)	7 (35)	8 (36)	32 (41)	11 (37)	3 (20)	9 (27)	7 (54)
Unacceptable	79 (14)	3 (15)	7 (32)	17 (22)	9 (30)	7 (47)	11 (33)	4 (31)
Very unacceptable	64 (12)	2 (10)	2 (9)	9 (11)	2 (7)	1 (7)	1 (3)	1 (8)
Don't know	262 (47)	5 (25)	1 (5)	16 (20)	8 (27)	2 (13)	10 (30)	1 (8)

BBS, bladder and bowel specialist.

### Facilitators and barriers to improving continence: HPs’ perspectives

HPs from all roles reported that funding frequently adversely affected provision of continence services. Many reported the lack of a dedicated continence service or a failure to provide a specific service in their area that was known to be available elsewhere. Ninety-one percent (142 of 156) of HPs reported lack of funding and/or resources as barriers to provision of dedicated continence services (table 5).

**Table 5:** Health professionals’ perspectives on: (a) the extent to which local commissioning/funding arrangements influence the toileting support they offer for children and young people with neurodisability; (b) whether where they work, there is a bladder and bowel protocol/pathway for children and young people with neurodisability; (c) whether there are any toileting interventions for children and young people with neurodisability that are not provided, which they know are provided elsewhere; (d) what they think is the main barrier to developing a dedicated bladder and bowel pathway or service

Question	Response option, n (% of respondents in role)	BBS nurses	Nurses	Paediatricians	Surgeons	Therapists	Other
(a) To what extent do local commissioning/funding arrangements influence the toileting support that you offer?		**N=22**	**N=79**	**N=31**	**N=15**	**N=33**	**N=13**
	Never	5 (23)	12 (15)	2 (6)	2 (13)	6 (18)	1 (8)
	Sometimes	6 (27)	18 (23)	10 (32)	5 (33)	8 (24)	5 (38)
	Often	3 (14)	11 (14)	10 (32)	4 (27)	5 (15)	1 (8)
	Always	6 (27)	19 (24)	6 (19)	1 (7)	1 (3)	1 (8)
	Don’t know	2 (9)	19 (24)	3 (10)	3 (20)	13 (39)	5 (38)
(b) Is there a bladder and bowel protocol/pathway?		**N=22**	**N=79**	**N=30**	**N=15**	**N=33**	**N=13**
	Yes	17 (77)	46 (58)	10 (33)	10 (67)	5 (15)	5 (38)
	No	4 (18)	21 (27)	17 (57)	4 (27)	13 (39)	4 (31)
	Don’t know	1 (5)	12 (15)	3 (10)	1 (7)	15 (45)	4 (31)
(c) Are there any toileting interventions that are not provided, which they know are provided elsewhere?		**N=22**	**N=79**	**N=30**	**N=15**	**N=33**	**N=13**
	Yes	6 (27)	18 (23)	8 (27)	6 (27)	18 (23)	8 (27)
	No	11 (50)	26 (33)	4 (13)	11 (50)	26 (33)	4 (13)
	Don’t know	5 (23)	35 (44)	18 (60)	5 (23)	35 (44)	18 (60)
(d) What is the main barrier to developing a dedicated bladder and bowel pathway or service?		**N=21**	**N=67**	**N=27**	**N=14**	**N=18**	**N=9**
	Lack of funding and/or resources	16 (76)	38 (57)	17 (63)	16 (76)	38 (57)	17 (63)
	Lack of professional interest	1 (5)	9 (13)	2 (7)	1 (5)	9 (13)	2 (7)
	Time	0 (0)	10 (15)	3 (11)	0 (0)	10 (15)	3 (11)
	Lack of need in local area	0 (0)	1 (1)	2 (7)	0 (0)	1 (1)	2 (7)
	Don’t know	1 (5)	6 (9)	1 (4)	1 (5)	6 (9)	1 (4)
	Other	3 (14)	3 (4)	2 (7)	3 (14)	3 (4)	2 (7)

Responses to (d) are shown for those health professionals who answered either ‘yes’ or ‘no’ to the question of whether there is a bladder and bowel protocol/pathway for children and young people with neurodisability—those who answered ‘don’t know’ are excluded.

BBS, bladder and bowel specialist.

HP perspectives varied on whether provision of pads/nappies was a barrier or enabler to toilet training. Excluding those who did not know, 16 of 20 BBS nurses, approximately 2 in 3 nurses, just over half of therapists, half of ‘other’ professionals, 7 of 17 paediatricians and 1 of 7 surgeons considered it a barrier (table 6). Reasons included:

*The continence pads don’t allow the child to feel wet therefore this reduces their understanding of doing wees and poos.* (Nurse 49013158)

*For some children it is a barrier as they become dependent on the security of the product and are less motivated to try toileting programmes. This is also age dependent - more successful when the toileting programmes are started earlier (chronological age rather than developmental age)*. (Nurse 49176801)

**Table 6:** Health professionals’ perspectives on whether provision of continence pads for children and young people with special educational needs and/or a disability is a barrier or enabler for achieving continence

Response option, n (% of respondents in role)	BBS nurses	Nurses	Paediatricians	Surgeons	Therapists	Other
	**N=22**	**N=76**	**N=30**	**N=15**	**N=33**	**N=13**
Barrier	16 (73)	39 (51)	7 (23)	1 (7)	9 (27)	5 (38)
Enabler	4 (18)	18 (24)	10 (33)	6 (40)	7 (21)	5 (38)
Don't know	2 (9)	19 (25)	13 (43)	8 (53)	17 (52)	3 (23)

BBS, bladder and bowel specialist.

## Discussion

This study explored the use, availability and acceptability of continence interventions for children and young people with neurodisability. Difficulties with implementing interventions identified by parent carers were consistent across clinical groups, reporting that the child was not ready, unable to learn or had inadequate understanding. SSC reported capability and time of the parent carer and lack of consistency of support across environments as key difficulties. Similarly, an earlier study has highlighted the importance of parent training as a key aspect of toilet training.^14^ The lack of adequate and accessible public facilities, highlighted by respondents as a barrier to toileting, emphasises the need for more accessible and/or specialist toilets; ‘Changing Places’ toilets are an example which provide necessary equipment and appropriate space in a clean and safe environment to support people with physical disabilities when outside home.^14^

Children and young people were generally unhappy about using many toileting interventions, particularly diets or catheter/bowel washouts. They also reported that using the toilet at home was preferable to toilets in other environments, perhaps due to inadequacy of other facilities as highlighted by SSC, and the important role the home plays in providing privacy for children and young people with disabilities.^15^ A review by Brazzelli *et al* also emphasised how medical or behavioural interventions can be upsetting for children and young people and therefore acceptability should be a key consideration in provision. Early introduction of these interventions has been shown to significantly improve acceptability to both children and young people and parents,^16^ and as many children and young people with neurodisability may not reach a level of understanding or willingness or reach a level of developmental ability to allow them to achieve continence, early education of parents and introduction of continence strategies is important. Our survey results only touched on acceptability of interventions, and this is an area that warrants further research.^17^

In contrast to the lack of acceptability of interventions indicated by children and young people, SSC said that diet and fluid intake advice, behavioural interventions, physical aids and continence products were easy to implement. Parent carers and SSC also reported continence products as easy to use, and HPs reported that they were typically provided free of charge, as recommended in National Health Service guidelines on managing incontinence in children with neurological disability.^18^ Some HPs, SSC and parent carers, however, did indicate that products were not free in their area, with many HPs also suggesting that provision of products was a barrier to achieving continence. Containment products do not necessarily improve continence in the same way as medical or behavioural-based toilet training interventions do, which aim for the child to become ‘clean and dry’. Impairment of children and young people with spinal cord pathology however may mean achieving continence is not a realistic goal and therefore the use of continence products is necessary, which can be at a considerable cost if not provided through health services.^10 18^ Recent research highlighted that provision of continence pads is inadequate and ad hoc,^19 20^ perhaps due to the influence that commissioning and funding arrangements have on provision, as highlighted in the HP survey. Research has also shown that children in low-income families are four times more likely to be urinary incontinent than children in high-income families,^21^ so those families who cannot afford continence products more likely experience compromised comfort and dignity.

Waiting times and funding were highlighted as influencing implementation of interventions. HPs had divided opinions on acceptability of waiting times to receive support and reported a lack of funding and dedicated services impacting provision. Similarly, parent carers reported variable waiting times and described difficulties accessing appropriate support. This is a key finding as coupled with a lack of continence product provision, it likely impacts the quality of life of the child and their family; similar findings were highlighted in studies of paediatric referrals for toileting support.^22 23^ Research by Kroeger and Sorensen-Burnworth^24^ emphasised that incontinence is a significant limiter of quality of life for those with developmental disabilities and difficulties in accessing adequate support in a timely manner are likely to significantly amplify the impact. Early intervention programmes can enable children to learn and develop age-appropriate toileting skills; however, age-related criteria in many areas for referral mean that opportunities for early intervention may be missed.

Strengths of an online survey methodology included the potential to reach a large, geographically and demographically diverse sample, at low cost and ease for participants, as demonstrated in other studies.^25 26^ Our surveys were developed and piloted with a diverse range of relevant stakeholders, parent carers and young people with neurodisability, ensuring that questions were relevant. Limitations included: the registration process led to potential delays in responses and risk of participants entering contact details incorrectly; partial response, perhaps due to the length of the survey; and responses were limited to those willing and able to use an online method. While there was an option for children and young people to have help to complete their survey, there was still a requirement for them to be able to express their feelings/experiences, meaning that only those with sufficient developmental ability to self-report were captured. Parent carers were not asked in the survey to report if their child had sufficient developmental capacity to adhere to an intervention. Therefore, where parent carers have reported their child’s lack of willingness as a difficulty in helping them to use toileting methods, it is not possible to distinguish whether the child they are reporting about has sufficient developmental capacity to be willing or not. Other limitations included that although data presented are from across England, the South West is over-represented, with this region having the largest proportion of respondents out of all of the regions, for all surveys. The survey findings may not adequately reflect variation in service provision and experience by locality, so can therefore only provide a snapshot of current practice. Overall, 93% of parent carers and children and young people who completed the surveys identified themselves as white, which is higher than the current census data in which 81.7% of usual residents in England and Wales identified as being in the white ethnic category.^27^ Finally, surveys were completed prior to the COVID-19 pandemic, so findings may be different when restrictions placed on health services during the pandemic are considered.

The survey findings highlighted perceived ability of the child and lack of adequate facilities as key factors affecting implementation of toileting interventions for children and young people with neurodisability. This emphasises the requirement of proactive and timely individual assessments of need, to facilitate the introduction of an appropriate individualised toileting skills development programme, to give the children and young people who have the appropriate physical and developmental ability the opportunity to become toilet trained or at least clean and dry at the same age as their typically developing peers. Previous guidance and research have also highlighted this need.^24 28^

The impact of incontinence on quality of life has previously been highlighted^24 29^ and our findings reinforce the need for further research around availability and acceptability of toileting interventions to adequately support children and young people with neurodisability. Effectiveness of interventions is likely to impact opinions of acceptability; while the surveys gathered some information on perceived effectiveness,^10^ further evidence would add to the knowledge around appropriate incontinence support for children and young people with neurodisability.

## Supplementary Material

Reviewer comments

Author's manuscript

## Data Availability

Data are available upon reasonable request. The full report of all survey findings is available from Open Research Exeter (ORE), https://ore.exeter.ac.uk/repository/handle/10871/127283
