# Supplementary material for: Interventions to improve continence for children and young people with neurodisability: a national survey of practitioner and family perspectives and experiences
Source: BMJ Paediatr Open. 2024 Jan 19;8(1):e002238. doi: 10.1136/bmjpo-2023-002238 (PMC10806478; doi:10.1136/bmjpo-2023-002238)
Supplement: Supplementary data [file bmjpo-2023-002238supp001.pdf]

Recruiting now!

We are conducting an online survey about toileting with health professionals, parent/carers, young people and education and social care staff.

The NHS wants to improve toileting services for children and young people with special educational needs and disability aged up to 25 years. We need to gather the views and experiences of families, clinicians, and school and social care staff, to establish how toileting is managed currently. The results of the survey will help inform health and care services for children in the future.

For further information and to take part in the survey, visit:
http://sites.exeter.ac.uk/iconstudy/taking-part/

ICoN
improving continence for children and young people with neurodisability

FUNDED BY NIHR | National Institute for Health Research
