# Supplementary material for: Interventions to improve continence for children and young people with neurodisability: a national survey of practitioner and family perspectives and experiences
Source: BMJ Paediatr Open. 2024 Jan 19;8(1):e002238. doi: 10.1136/bmjpo-2023-002238 (PMC10806478; doi:10.1136/bmjpo-2023-002238)
Supplement: Supplementary data [file bmjpo-2023-002238supp003.pdf]

Supplementary Material Table 1: Parent carers' and school and social care staff's perspectives on how easy specified toileting methods are to use at home, and how easy it is for them to provide or use specified methods to help children and young people with toileting, respectively

**Survey respondents / clinical group**	**Method, n (% of respondents)**	**How easy it is to use the method**				
		**Very easy**	**Easy**	**Difficult**	**Very difficult**	**Never used**
**Parent carers / non-spinal cord pathology**	Dietary advice (N = 476) *(N = 244)*	37 (8) *(15)*	104 (22) *(43)*	64 (13) *(26)*	39 (8) *(16)*	232 (49)
	Fluid intake advice (N = 491) *(N = 324)*	51 (10) *(16)*	140 (29) *(43)*	101 (21) *(31)*	32 (7) *(10)*	167 (34)
	Behavioural intervention e.g., reward chart, timer, alarm (N = 483) *(N = 292)*	18 (4) *(6)*	72 (15) *(25)*	99 (20) *(34)*	103 (21) *(35)*	191 (40)
	Simple aid/equipment e.g., raised step or seat (N = 479) *(N = 292)*	60 (13) *(21)*	156 (33) *(53)*	54 (11) *(18)*	22 (5) *(8)*	187 (39)
	Bespoke aid/equipment e.g., hoist or frame (N = 456) *(N = 77)*	12 (3) *(16)*	31 (7) *(40)*	22 (5) *(29)*	12 (3) *(16)*	379 (83)
	Housing adaptions e.g., specialised toilet (N = 456) *(N = 68)*	16 (4) *(24)*	23 (5) *(34)*	15 (3) *(22)*	14 (3) *(21)*	388 (85)
	Continence products e.g., nappies, pads, pull ups (N = 497) *(N = 401)*	132 (27) *(33)*	188 (38) *(47)*	60 (12) *(15)*	21 (4) *(5)*	96 (19)
	Medication e.g., laxatives, anticholinergics, desmopressin (N = 489) *(N = 295)*	55 (11) *(19)*	131 (27) *(44)*	73 (15) *(25)*	36 (7) *(12)*	194 (40)
	Catheters (N = 442) *(N = 14)*	2 (<1) *(14)*	3 (<1) *(21)*	5 (1) *(36)*	4 (1) *(29)*	428 (97)
	Colonic enema (bowel washout) (N = 450) *(N = 29)*	4 (1) *(14)*	11 (2) *(38)*	3 (<1) *(10)*	11 (2) *(38)*	421 (94)

	Surgical intervention e.g., botox, bladder reconstruction, mitrofanoff (N = 447) *(N = 19)*	1	(<1) *(5)*	6	(1) *(32)*	4	(1) *(21)*	8	(2) *(42)*	428	(96)
		**Very easy**		**Easy**		**Difficult**		**Very difficult**		**Never used**	
**Parent carers / spinal cord pathology**	Dietary advice (N = 18) *(N = 11)*	5	(28) *(45)*	2	(11) *(18)*	4	(22) *(36)*	0	(0) *(0)*	7	(39)
	Fluid intake advice (N = 18) *(N = 13)*	4	(22) *(31)*	3	(17) *(23)*	5	(28) *(38)*	1	(6) *(8)*	5	(28)
	Behavioural intervention e.g., reward chart, timer, alarm (N = 17) *(N = 7)*	0	(0) *(0)*	3	(18) *(43)*	2	(12) *(29)*	2	(12) *(29)*	10	(59)
	Simple aid/equipment e.g., raised step or seat (N = 20) *(N = 12)*	5	(25) *(42)*	3	(15) *(25)*	2	(10) *(17)*	2	(10) *(17)*	8	(40)
	Bespoke aid/equipment e.g., hoist or frame (N = 18) *(N = 4)*	1	(6) *(25)*	1	(6) *(25)*	2	(11) *(50)*	0	(0) *(0)*	14	(78)
	Housing adaptions e.g., specialised toilet (N = 19) *(N = 5)*	2	(11) *(40)*	3	(16) *(60)*	0	(0) *(0)*	0	(0) *(0)*	14	(74)
	Continence products e.g., nappies, pads, pull ups (N = 19) *(N = 16)*	7	(37) *(44)*	8	(42) *(50)*	1	(5) *(6)*	0	(0) *(0)*	3	(16)
	Medication e.g., laxatives, anticholinergics, desmopressin (N = 18) *(N = 17)*	6	(33) *(35)*	4	(22) *(24)*	4	(22) *(24)*	3	(17) *(18)*	1	(6)
	Catheters (N = 19) *(N = 15)*	4	(21) *(27)*	8	(42) *(53)*	2	(11) *(13)*	1	(5) *(7)*	4	(21)
	Colonic enema (bowel washout) (N = 18) *(N = 11)*	1	(6) *(9)*	6	(33) *(55)*	1	(6) *(9)*	3	(17) *(27)*	7	(39)
	Surgical intervention e.g., botox, bladder reconstruction, mitrofanoff (N = 18) *(N = 6)*	2	(11) *(33)*	2	(11) *(33)*	1	(6) *(17)*	1	(6) *(17)*	12	(67)

		Very easy		Easy		Difficult		Very difficult		Never used	
**School and social care staff / not separated by clinical group**	Dietary advice (N = 116) *(N = 96)*	17	(15) *(18)*	59	(51) *(61)*	19	(16) *(20)*	1	(1) *(1)*	20	(17)
	Fluid intake advice (N = 116) *(N = 106)*	24	(21) *(23)*	64	(55) *(60)*	16	(14) *(15)*	2	(2) *(2)*	10	(9)
	Behavioural intervention e.g., reward charts, timers, alarms (N = 115) *(N = 109)*	46	(40) *(42)*	52	(45) *(48)*	10	(9) *(9)*	1	(1) *(1)*	6	(5)
	Physical aids / equipment e.g., specialised toilets, hoists, frames (N = 116) *(N = 99)*	30	(26) *(30)*	47	(41) *(47)*	15	(13) *(15)*	7	(6) *(7)*	17	(15)
	Continence products e.g., nappies, pads, pull ups, continence pants (N = 114) *(N = 102)*	34	(30) *(33)*	49	(43) *(48)*	15	(13) *(15)*	4	(4) *(4)*	12	(11)
	Medication or drugs e.g., laxatives, anticholinergics, desmopressin (N = 113) *(N = 72)*	15	(13) *(21)*	37	(33) *(51)*	19	(17) *(26)*	1	(1) *(1)*	41	(36)
	Catheterisation (N = 113) *(N = 31)*	8	(7) *(26)*	11	(10) *(35)*	9	(8) *(29)*	3	(3) *(10)*	82	(73)
	Colonic enema (bowel washout) (N = 112) *(N = 21)*	1	(1) *(5)*	5	(4) *(24)*	12	(11) *(57)*	3	(3) *(14)*	91	(81)
	Surgical intervention e.g., cytoscopic botox, ACE, bladder reconstruction, mitrofanoff (N = 109) *(N = 16)*	2	(2) *(13)*	4	(4) *(25)*	8	(7) *(50)*	2	(2) *(13)*	93	(85)

Numbers in italic show the total number of respondents (*N*) and percentage of respondents choosing the response option, excluding those who answered ‘Never used’. ACE = antegrade colonic enema.

Supplementary Material Table 2: Health professionals', school and social care staff's, and parent carers' perspectives on provision of interventions to families in their area

**Response option, n (% of respondent group)**	**Health professionals**						**School and social care staff**	**Parent carers**	
	**BBS Nurses**	**Nurses**	**Paediatricians**	**Surgeons**	**Therapists**	**Other**		**Non-spinal cord pathology**	**Spinal cord pathology**
**Behavioural - e.g., timer, alarm**	**N = 20**	**N = 74**	**N = 29**	**N = 15**	**N = 27**	**N = 12**	**N = 112**	**N = 519**	**N = 17**
Free of charge	12 (60)	26 (35)	14 (48)	5 (33)	5 (19)	4 (33)	22 (20)	29 (6)	0 (0)
Subsidised	0 (0)	0 (0)	2 (7)	0 (0)	1 (4)	1 (8)	3 (3)	1 (<1)	0 (0)
Available to purchase	0 (0)	10 (14)	0 (0)	1 (7)	4 (15)	0 (0)	18 (16)	46 (9)	0 (0)
Unavailable	6 (30)	26 (35)	4 (14)	6 (40)	11 (41)	6 (50)	14 (13)	49 (9)	2 (12)
Don't know (/ never used)**	2 (10)	12 (16)	9 (31)	3 (20)	6 (22)	1 (8)	55 (49)	394 (76)	15 (88)
**Simple aid/equipment - e.g., raised seat or step**	**N = 18**	**N = 74**	**N = 29**	**N = 15**	**N = 27**	**N = 12**	**N = 113***	**N = 523**	**N = 19**
Free of charge	7 (39)	27 (36)	11 (38)	2 (13)	20 (74)	3 (25)	43 (38)	91 (17)	8 (42)
Subsidised	1 (6)	1 (1)	0 (0)	1 (7)	0 (0)	0 (0)	7 (6)	2 (<1)	0 (0)
Available to purchase	2 (11)	12 (16)	7 (24)	1 (7)	1 (4)	1 (8)	18 (16)	69 (13)	2 (11)
Unavailable	5 (28)	25 (34)	3 (10)	6 (40)	3 (11)	5 (42)	13 (12)	45 (9)	2 (11)
Don't know (/ never used)**	3 (17)	9 (12)	8 (28)	5 (33)	3 (11)	3 (25)	32 (28)	316 (60)	7 (37)
**Bespoke aid/equipment - e.g., hoist or frame**	**N = 18**	**N = 74**	**N = 29**	**N = 15**	**N = 27**	**N = 12**		**N = 521**	**N = 17**
Free of charge	9 (50)	42 (57)	18 (62)	0 (0)	20 (74)	2 (17)		73 (14)	2 (12)

Subsidised	2	(11)	2	(3)	0	(0)	1	(7)	1	(4)	1	(8)			5	(1)	0	(0)
Available to purchase	1	(6)	3	(4)	0	(0)	0	(0)	2	(7)	0	(0)			12	(2)	0	(0)
Unavailable	3	(17)	15	(20)	1	(3)	6	(40)	1	(4)	4	(33)			20	(4)	0	(0)
Don't know (/ never used)**	3	(17)	12	(16)	10	(34)	8	(53)	3	(11)	5	(42)			411	(79)	15	(88)
**Housing adaption - e.g., specialised toilet**	**N = 18**		**N = 72**		**N = 29**		**N = 15**		**N = 27**		**N = 12**		**N = 113**		**N = 516**		**N = 19**	
Free of charge	7	(39)	26	(36)	15	(52)	0	(0)	13	(48)	3	(25)	29	(26)	61	(12)	5	(26)
Subsidised	3	(17)	5	(7)	3	(10)	1	(7)	4	(15)	1	(8)	14	(12)	19	(4)	1	(5)
Available to purchase	1	(6)	4	(6)	0	(0)	0	(0)	2	(7)	0	(0)	9	(8)	14	(3)	1	(5)
Unavailable	3	(17)	14	(19)	1	(3)	6	(40)	4	(15)	4	(33)	11	(10)	20	(4)	0	(0)
Don't know (/ never used)**	4	(22)	23	(32)	10	(34)	8	(53)	4	(15)	4	(33)	50	(44)	402	(78)	12	(63)
**Products - e.g., pads, nappies, continence pants, pull ups**	**N = 18**		**N = 72**		**N = 29**		**N = 14**		**N = 26**		**N = 12**		**N = 115**		**N = 534**		**N = 19**	
Free of charge	17	(94)	53	(74)	19	(66)	8	(57)	11	(42)	6	(50)	55	(48)	256	(48)	9	(47)
Subsidised	1	(6)	5	(7)	4	(14)	0	(0)	4	(15)	1	(8)	8	(7)	21	(4)	1	(5)
Available to purchase	0	(0)	2	(3)	1	(3)	0	(0)	0	(0)	0	(0)	17	(15)	55	(10)	6	(32)
Unavailable	0	(0)	7	(10)	0	(0)	3	(21)	2	(8)	4	(33)	13	(11)	42	(8)	1	(5)
Don't know (/ never used)**	0	(0)	5	(7)	5	(17)	3	(21)	9	(35)	1	(8)	22	(19)	160	(30)	2	(11)
**Medications - e.g., laxatives**	**N = 18**		**N = 72**		**N = 29**		**N = 14**		**N = 26**		**N = 12**		**N = 113**		**N = 522**		**N = 18**	
Free of charge	15	(83)	60	(83)	29	(100)	13	(93)	8	(31)	9	(75)	47	(42)	290	(56)	17	(94)
Subsidised	1	(6)	0	(0)	0	(0)	1	(7)	0	(0)	0	(0)	2	(2)	3	(<1)	0	(0)
Available to purchase	0	(0)	0	(0)	0	(0)	0	(0)	0	(0)	1	(8)	7	(6)	15	(3)	0	(0)

Unavailable	1	(6)	10	(14)	0	(0)	0	(0)	2	(8)	2	(17)	15	(13)	6	(1)	0	(0)
Don't know (/ never used)**	1	(6)	2	(3)	0	(0)	0	(0)	16	(62)	0	(0)	42	(37)	208	(40)	1	(6)

* = Aids/equipment not separated into simple or bespoke in school and social care survey; ** = response option includes *never used* for school and social care staff and parent carer surveys; BBS = Bladder and Bowel Specialist.
