# Supplementary material for: Interventions to improve continence for children and young people with neurodisability: a national survey of practitioner and family perspectives and experiences
Source: BMJ Paediatr Open. 2024 Jan 19;8(1):e002238. doi: 10.1136/bmjpo-2023-002238 (PMC10806478; doi:10.1136/bmjpo-2023-002238)
Supplement: Author's manuscript [file bmjpo-2023-002238.draft_revisions.pdf]

BMJ Paediatrics Open

BMJ Paediatrics Open is committed to open peer review. As part of this commitment we make the peer review history of every article we publish publicly available.

When an article is published we post the peer reviewers' comments and the authors' responses online. We also post the versions of the paper that were used during peer review. These are the versions that the peer review comments apply to.

The versions of the paper that follow are the versions that were submitted during the peer review process. They are not the versions of record or the final published versions. They should not be cited or distributed as the published version of this manuscript.

BMJ Paediatrics Open is an open access journal and the full, final, typeset and author-corrected version of record of the manuscript is available on our site with no access controls, subscription charges or pay-per-view fees (http://bmjpaedsopen.bmj.com).

If you have any questions on BMJ Paediatrics Open's open peer review process please email info.bmjpo@bmj.com

BMJ Paediatrics Open

Interventions to improve continence for children and young people with neurodisability: a national survey of practitioner and family perspectives and experiences

Journal:	*BMJ Paediatrics Open*
Manuscript ID	bmjpo-2023-002238
Article Type:	Original research
Date Submitted by the Author:	18-Aug-2023

Complete List of Authors:	Eke, Helen; University of Exeter, Medical School<br>Ball, Susan; University of Exeter, Medical School<br>Allinson, Annette; University of Exeter, Peninsula Childhood Disability Research Unit<br>Anderson, Rob; University of Exeter, Medical School<br>Hunt, Harriet; University of Exeter, Medical School<br>Hutton, Eve; Canterbury Christ Church University<br>Lindsay, Claire; Northern Devon Healthcare NHS Trust<br>Logan, Stuart; University of Exeter, Child Health, Medical School<br>Madden, Nicholas; Chelsea and Westminster Hospital NHS Foundation Trust<br>Melluish, Julia; University of Exeter, Peninsula Childhood Disability Research Unit<br>Richardson, Davina; Disabled Living Foundation<br>Rogers, June; Disabled Living Foundation<br>Thompson Coon, Jo; University of Exeter, National Institute for Health Research (NIHR) Applied Research Collaboration (ARC) South West Peninsula (PenARC)<br>Whear, Rebecca; University of Exeter, National Institute for Health Research (NIHR) Applied Research Collaboration (ARC) South West Peninsula (PenARC)<br>Wright, Anne; Guy's and St Thomas' Hospitals NHS Trust<br>Morris, Christopher; University of Exeter, Child Health, Medical School

Keywords:	Neurodisability, Enuresis, Health services research

SCHOLARONE™
Manuscripts

I, the Submitting Author has the right to grant and does grant on behalf of all authors of the Work (as defined in the below author licence), an exclusive licence and/or a non-exclusive licence for contributions from authors who are: i) UK Crown employees; ii) where BMJ has agreed a CC-BY licence shall apply, and/or iii) in accordance with the terms applicable for US Federal Government officers or employees acting as part of their official duties; on a worldwide, perpetual, irrevocable, royalty-free basis to BMJ Publishing Group Ltd ("BMJ") its licensees and where the relevant Journal is co-owned by BMJ to the co-owners of the Journal, to publish the Work in this journal and any other BMJ products and to exploit all rights, as set out in our licence.

The Submitting Author accepts and understands that any supply made under these terms is made by BMJ to the Submitting Author unless you are acting as an employee on behalf of your employer or a postgraduate student of an affiliated institution which is paying any applicable article publishing charge ("APC") for Open Access articles. Where the Submitting Author wishes to make the Work available on an Open Access basis (and intends to pay the relevant APC), the terms of reuse of such Open Access shall be governed by a Creative Commons licence – details of these licences and which Creative Commons licence will apply to this Work are set out in our licence referred to above.

Other than as permitted in any relevant BMJ Author's Self Archiving Policies, I confirm this Work has not been accepted for publication elsewhere, is not being considered for publication elsewhere and does not duplicate material already published. I confirm all authors consent to publication of this Work and authorise the granting of this licence.

Confidential: For Review Only

Interventions to improve continence for children and young people with neurodisability: a national survey of practitioner and family perspectives and experiences

Eke H[1,2*†], Ball S[1*], Allinson A[2], Anderson R[3], Hunt H[3], Hutton E[4], Lindsay C[5], Logan S[1,2], Madden N[6], Melluish J[2], Richardson D[7], Rogers J[7], Thompson Coon J[1,3], Whear R[1,3], Wright A[8], Morris C[1,2]

*Equal contribution; †Corresponding author h.e.eke@exeter.ac.uk ORCID 0000-0003-4781-6683

[1] National Institute for Health Research (NIHR) Applied Research Collaboration (ARC) South West Peninsula (PenARC), Exeter, UK

[2] Peninsula Childhood Disability Research Unit (PenCRU), University of Exeter Medical School, University of Exeter, Exeter, UK

[3] Evidence Synthesis & Modelling for Health Improvement (ESMI), University of Exeter Medical School, University of Exeter, Exeter, UK

[4] School of Allied Health Professions, Canterbury Christ Church University, Canterbury, UK

[5] Paediatric Bladder and Bowel Care Team, Northern Devon Healthcare NHS Trust, Exeter, UK

[6] Children's Surgery, Chelsea and Westminster Hospital NHS Foundation Trust, London, UK

[7] Bladder & Bowel UK, Disabled Living, Manchester, UK

[8] Evelina London Children's Hospital, Guy's and St Thomas' NHS Foundation Trust, London, UK

Word count [text excluding refs 3077 / 2500]

https://mc.manuscriptcentral.com/bmjpo

Confidential: For Review Only

Abstract [283/300 words]

Objective: Describe families' experiences of interventions to improve continence in children and young people with neurodisability, and health professionals' and school and social care staff's perspectives regarding factors affecting intervention use.

Design: Four online surveys were developed and advertised to parent carers, young people with neurodisability, health professionals and school and social care staff, via societies, charities, professional contacts, schools, local authorities, and national parent carer and family forums, who shared invitations with their networks. Survey questions explored: difficulties helping children and young people use interventions; acceptability of interventions and waiting times; ease of use and availability of interventions, and facilitators and barriers to improving continence.

Results: 1028 parent carers, 26 young people, 352 health professionals and 202 school and social care staff registered to participate. Completed surveys were received from 579 (56.3%) parent carers, 20 (77%) young people, 193 (54.8%) health professionals, and 119 (58.9%) school and social care staff. Common parent carer-reported difficulties in using interventions to help their children and young people to learn to use the toilet included their child's lack of understanding about what was required (reported by 337/556 (60.6%) parent carers who completed question) and their child's lack of willingness (343/556, 61.7%). Almost all (142/156, 91%) health professionals reported lack of funding and resources as barriers to provision of continence services. Many young people (14/19, 74%) were unhappy using toilet facilities whilst out and about.

Conclusions: Perceptions that children lack understanding and willingness, and inadequate facilities, impact the implementation of toileting interventions for children and young people with neurodisability. Greater understanding is needed for children to develop age-appropriate toileting skills. Further research is recommended around availability and acceptability of interventions to ensure quality of life is unaffected.

https://mc.manuscriptcentral.com/bmjpo

For Review Only

What is already known on this topic

- Children with neurodisability are often delayed in acquiring continence, and are more frequently incontinent than typically developing children. Social, economic, and environmental factors, parenting strategies and child behaviour all affect toilet training.
- Incontinence can affect the quality of life of the child or young person and their carers.
- Many children with neurodisability can become continent, and, while not all children are able to become fully independent in toileting, many can improve their continence with support, with consequent positive gains in quality of life.
- A variety of approaches and interventions are available including information and support, charts to monitor/feedback, schedules for drinking and toileting, cognitive behavioural approaches, equipment, aids, relaxation techniques, psychotherapy, group-based programmes, medicines, and surgery. Previous research has identified limited evidence for toilet training strategies for children with physical and learning disabilities.

What this paper adds

- Families describe inadequate access to continence support and interventions.
- Many children are perceived as lacking willingness and understanding regarding continence needs.
- Many health professionals reported poor local availability of some interventions, and a lack of funding for dedicated continence services.
- Children and young people were unhappy with some interventions and with out-of-home toilet facilities.

How this study might affect research, practice or policy

- Highlights the need for further research around acceptability of interventions to improve continence in children and young people with neurodisability, without compromising quality of life.
- Emphasises the requirement for an individual assessment of needs for children and young people with neurodisability, to be carried out proactively and in a timely manner.
- Funding issues are likely restricting the toileting interventions that are being offered to children and young people with neurodisability.

Confidential: For Review Only

Introduction

Neurodisability describes a group of congenital or acquired long-term conditions attributed to the impairment of the brain and/or neuromuscular system, creating functional limitations including difficulties with movement, cognition, hearing, vision, communication, emotion, and behaviour (1). Children with neurodisability are often delayed in acquiring continence, and more frequently incontinent than typically developing children; they may be slower to learn to manage toileting or may need additional support (1–3). Social, economic, and environmental factors, parenting strategies and child behaviour can all affect toilet training.

Continence is being clean and dry without using containment products such as pads/nappies. Incontinence can affect the quality of life of the young person and their carers (4) and the long-term physical, psychological and financial burden can be considerable (5). Many children with neurodisability can become continent, and, while not all are able to become fully independent in toileting, many can improve their continence with support (2–4), with consequent improvement in quality of life.

Distinguishing continence for individuals with and without spinal cord pathology affecting bladder and bowel sensorimotor control is crucial. Without sensation and motor control, and normal detrusor, colonic and sphincter function, assistive technology or alternative approaches to bladder and bowel storage and emptying will often be needed.

A variety of approaches and interventions are available (6) including information and support, monitoring/feedback charts, drinking and toileting schedules, cognitive behavioural approaches, equipment, aids, relaxation techniques, psychotherapy, group-based programmes, medicines and surgery (7). An existing systematic review identified limited evidence for toilet training strategies for children with physical and learning disabilities (8).

Research to evaluate ways to promote continence for children with neurodisability ranked 7/10 in the British Academy of Childhood Disability James Lind Alliance Research Priority Setting Partnership (9). Subsequently, the ICoN (Improving Continence in children and young people with Neurodisability) study was commissioned by the National Institute for Health and Care Research to summarise available evidence for interventions and practice relating to improving continence for children and young people with neurodisability (10). Online surveys were conducted with parent carers, children and young people with neurodisability, health professionals and school and social care staff. This paper details the survey process and aims to describe the experiences and perspectives of parent carers and children and young people

Confidential: For Review Only

with neurodisability regarding interventions to improve continence, and to explore health professionals' and school and social care staff's perspectives regarding factors affecting intervention use.

Confidential: For Review Only

Methods

Survey development

Survey questions and response options were developed in collaboration with the ICoN Family Faculty public involvement group, Professional Advisory Group, and young people with neurodisability. Surveys were developed through iteration and piloting.

The surveys included adaptive questioning; respondents were only asked to answer questions that were relevant to them. Parent carers, health professionals (HP) and school and social care staff (SSC) completed an initial eligibility question (related to their experience of continence support and neurodisability), and chose a clinical group about which to complete the survey; non-spinal cord pathology, defined as *social/communication or attention/behaviour difficulty, learning disability or physical or movement disability*, or spinal cord pathology, defined as *bladder and/or bowel impairment due to damage to the spinal cord e.g., neurogenic/neuropathic condition.* HPs had the option to complete the survey for both clinical groups.

Question order was fixed within surveys, following a logical order in terms of the respondent characteristics and experiences. Surveys included a maximum of five demographic characteristics questions, and up to 14 questions about professional practice and/or experience regarding interventions for improving continence. The children and young people (CYP) survey included just four questions regarding toileting abilities and experiences. The HP and SSC surveys included 16 additional questions regarding service provision, which all respondents, regardless of chosen clinical group, were asked to complete. HP survey questions reported in this paper come from the service provision section, not separated by clinical group. Box 1 lists the survey questions covered in this paper.

Participants and procedure

Parent carers, CYP, HPs, and SSC were invited to participate via a study advert, shared by over 100 societies, charities, organisations, professional contacts, schools, local authorities and national parent carer and family forums. Study information was also advertised regularly by the research team, via social media. A copy of the study advert can be found in the supplementary material. This targeted recruitment gathered a diverse sample of participants with knowledge and experience of continence services and interventions for CYP with neurodisability.

Participants registered via a study website (https://blogs.exeter.ac.uk/iconstudy/) to be emailed a personalised link to complete the relevant survey. This registration process created a 'closed'

Confidential: For Review Only

survey, allowing the study team to know to whom the invitations were sent, rather than an open survey for anyone to answer. Monitoring of registered email addresses allowed identification of duplicated registrations, and up to four reminders were sent to non-responders. Each participant was assigned a unique identifier via the registration website, preventing participants from completing the survey more than once. No analysis of IP addresses or log files was conducted.

Informed consent was assumed through voluntary registration online (11). Participants were encouraged to read the participant information sheet available via the registration webpage. Participation was voluntary, and no participation incentive was offered. The time required to complete the survey was not recorded although participants were advised that the survey required mostly tick box responses and would take approximately 15 minutes. Participants were able to review and save their responses prior to submission. All individually identifiable data were password protected via the registration website.

Data collection and reporting

The surveys were open between July and December 2019.

Numbers of registrations and completed surveys were recorded. Data were exported into Microsoft Excel and analysed using R (12). Guidelines for reporting online surveys were followed (13)), see supplementary material for a copy of the completed CHERRIES checklist. The number and percentage of completed surveys (as a proportion of personalised links sent) are reported. Demographic characteristics are summarised and the number of responses to each survey question reported. As not all questions were mandatory, the total number of responses varies across questions. Responses of ‘don’t know’, ‘never used’, ‘no knowledge’ or ‘never happened to me’ regarding a service or intervention, are excluded from the description of results but are included in results tables. Parent carer survey responses are reported by clinical group. HP survey responses are reported by job role. Selected free-text responses illustrate salient results from the numerical data.

Ethics approval

The University of Exeter Medical School Research Ethics Committee approved the procedures (UEMS REC 19/B/199).

Confidential: For Review Only

Results

Survey responses

In total, 605 responses were received from 1028 parent carer registrations, 20 from 26 registered CYP, 202 from 352 HP registrations, and 122 from 202 SSC registrations. Nine responses were excluded from both the parent carer and HP survey and three from the SSC survey due to ineligibility (no experience of continence support for, or the care of, CYP with neurodisability). A further 17 parent carer responses were excluded because their child did not belong to one of the two clinical groups (n = 10) or could not be allocated to one of these groups from their free-text description of their child's condition (n = 7). Completed survey responses are therefore reported from 579 (56.3%) parent carers, 20 (77%) CYP, 193 (54.8%) HPs, and 119 (58.9%) SSC.

Characteristics of respondents

Ninety percent (522/579) of parent carers were the child's mother, 396 (68.4%) of the children about whom the parent carers were completing the survey were male, and 446 (77%) were aged between 5 and 17 years. Over two thirds of CYP respondents were female (14/20), of white ethnic origin (19/20), aged over 18 years (14/20) and were identified as being in the non-spinal cord pathology clinical group (17/20) from a free-text description of their condition. HPs were grouped into six categories by job role: bladder and bowel specialist (BBS) nurse; nurse; paediatrician; surgeon; therapist; other. Details of the roles included within these categories are provided elsewhere (9). Common SSC roles were teachers (29/119, 24.4%), assistants (28/119, 23.5%) and 'others' (36/119, 30.3%), and almost half worked in special schools (51/119, 42.9%). All geographical regions of England were represented (Table 1).

Difficulties in helping children and young people use interventions: parent carers' and school and social care staff's perspectives

The pattern of responses from parent carers regarding difficulties in helping CYP use toileting methods was similar for both clinical groups. The most common difficulties reported were the child's lack of understanding (337/556, 60.6%) and willingness (343/556, 61.7%). Regarding services, the most frequently reported difficulties were access to appropriate help (240/556, 43.2%); lack of consistency of support in different environments (209/556, 37.6%); delays in professional assessments (151/556, 27.2%); and lack of funding for equipment and products (129/556, 23.2%). The most common SSC-reported difficulties included CYP's lack of

Confidential: For Review Only

understanding of what was required (67/115, 58.3%), limited parent carer capability and time (70/115, 60.9%), and lack of appropriate facilities at school (69/115, 60%) (Table 2).

Acceptability of interventions: children's and young people's perspectives

Most CYP with experience of toileting interventions felt happy or OK about using a hoist or frame (8/10), an alarm or timer (6/9), and half felt positively about using continence products and medications (6/12 for both). In contrast, many indicated unhappiness with following water/food diets (9/16), using a catheter or bowel washout (5/8) or having surgery (8/10). Most CYP felt happy or OK using the toilet at home (14/18), but many felt negatively about using toilets at school, college or work (8/18), and out and about (14/19) (Table 3).

Ease of use of interventions: parent carers' and school and social care staff's perspectives

Parent carers reported that toileting products (320/401, 80%) and simple aids/equipment (216/292, 74%) were easiest to use in the non-spinal cord pathology group. In the spinal cord pathology group, all five parent carers with experience of housing adaptations, and 15/16 with experience of continence products, reported them to be easy or very easy to use.

A large proportion of SSC with experience of interventions to promote continence found toileting products or training methods easy or very easy to use; dietary advice (76/96, 79%), fluid intake advice (88/106, 83%), and behavioural interventions (98/109, 89.9%). In contrast, only 31% of parent carers (93/299) found behavioural interventions easy or very easy to use. (Table 4).

Availability of interventions: parent carers', school and social care staff's and health professionals' perspectives

Substantial numbers of HPs with knowledge of specific aids, said they were not available or only available by purchase (behavioural aids such as alarms, 74/144 (51.4%); simple aids such as raised seats or steps, 71/146 (48.6%); and housing adaptations, 39/124 (31.4%)). The most common response regarding provision of continence products was that they were supplied free of charge (17/18, 94% bladder and bowel specialist (BBS) nurses; 11/17, 65% therapists; 55/93, 59% SSC; 256/374, 68.4% and 9/17, 53% parent carers for non-spinal cord and spinal cord pathologies, respectively) (Table 5).

Acceptability of waiting times for continence support: parent carers' and health professionals' perspectives

Confidential: For Review Only

Roughly half of parent carers and HPs with knowledge of waiting times for continence support considered them acceptable (Table 6). Parent carers and HPs who considered waiting times to be unacceptable, described long waits for assessments, lack of funding and staffing:

> *"Waiting times are too long for assessment, and then provision of complex equipment and/or adaptations can also take a long time". [Therapist 48880209]*
>
> *"Not enough continence support so waiting times long and services are not funded". [Nurse 49177881]*
>
> *"Everything takes a very long time to come through, including assessment - not had one for years". [Parent carer 49575343]*
>
> *"Two part time staff for the whole county, very few appointments and long waiting list. Very restrictive criteria to access the service". [Parent carer 50843591]*
>
> *"Funding is always an issue. It's always a fight to get appropriate services" [Parent carer 50044306]*

Facilitators and barriers to improving continence: health professionals' perspectives

HPs from all roles reported that funding frequently adversely affected provision of continence services. Many reported the lack of a dedicated continence service or a failure to provide a specific service in their area that was known to be available elsewhere. Ninety-one percent (142/156) of HPs reported lack of funding and/or resources as barriers to provision of dedicated continence services (Table 7).

HP perspectives varied on whether provision of pads/nappies was a barrier or enabler to toilet training. Excluding those who didn't know, 16/20 BBS nurses, approximately 2 in 3 nurses, just over half of therapists, half of 'other' professionals, 7/17 paediatricians and 1/7 surgeons, considered it a barrier (Table 8). Reasons included:

> *"The continence pads don't allow the child to feel wet therefore this reduces their understanding of doing wees and poos". [Nurse 49013158]*
>
> *"For some children it is a barrier as they become dependent on the security of the product and are less motivated to try toileting programmes. This is also age dependent - more successful when the toileting programmes are started earlier (chronological age rather than developmental age)". [Nurse 49176801]*

Confidential: For Review Only

Discussion

This study explored the use, availability and acceptability of continence interventions for children and young people with neurodisability. Difficulties with implementing interventions identified by parent carers were consistent across clinical groups, reporting that the child was not ready, unable to learn or had inadequate understanding. SSC reported capability and time of the parent carer and lack of consistency of support across environments as key difficulties. Similarly, an earlier study has highlighted the importance of parent training as a key aspect of toilet training (14). The lack of adequate and accessible public facilities highlighted by respondents as a barrier to toileting, emphasises the need for more accessible and/or specialist toilets; 'Changing Places' toilets are an example which provide necessary equipment and appropriate space in a clean and safe environment to support people with physical disabilities when outside home (14).

CYP were generally unhappy about using many toileting interventions, particularly diets or catheter/bowel washouts. They also reported that using the toilet at home was preferable to toilets in other environments, perhaps due to inadequacy of other facilities as highlighted by SSC, and the important role the home plays in providing privacy for CYP with disabilities (15). A review by Brazzelli et al (2011) also emphasised how medical or behavioural interventions can be upsetting for CYP and therefore acceptability should be a key consideration in provision. Early introduction of these interventions has been shown to significantly improve acceptability to both CYP and parents (16), and as many CYP with neurodisability may not reach a level of understanding or willingness, early education of parents and introduction of continence strategies is important. Our survey results only touched on acceptability of interventions, and this is an area that warrants further research (17).

In contrast to the lack of acceptability of interventions indicated by CYP, SSC said that diet and fluid intake advice, behavioural interventions, physical aids, and continence products were easy to implement. Parent carers and SSC also reported continence products as easy to use, and HPs reported that they were typically provided free of charge, as recommended in NHS guidelines on managing incontinence in children with neurological disability (18). Some HPs, SSC and parent carers, however, did indicate that products were not free in their area, with many HPs also suggesting that provision of products was a barrier to achieving continence. Containment products do not necessarily improve continence in the same way as medical or

Confidential: For Review Only

behavioural-based toilet training interventions do, which aim for the child to become 'clean and dry'. Impairment of CYP with spinal cord pathology however, may mean that continence product use is necessary, which can be at a considerable cost if not provided through health services (10,18). Recent research highlighted that provision of continence pads is inadequate and *ad hoc* (19,20), perhaps due to the influence that commissioning and funding arrangements have on provision, as highlighted in the HP survey.

Waiting times and funding were highlighted as influencing implementation of interventions. HPs had divided opinions on acceptability of waiting times to receive support and reported a lack of funding and dedicated services impacting provision. Similarly, parent carers reported variable waiting times, and described difficulties accessing appropriate support. This is a key finding as it likely impacts the quality of life of the child and their family; similar findings were highlighted in studies of paediatric referrals for toileting support (21,22). Research by Kroeger and Sorensen-Burnworth (23) emphasised that incontinence is a significant limiter of quality of life for those with developmental disabilities and difficulties in accessing adequate support in a timely manner is likely to significantly amplify the impact. Early intervention programmes can enable children to learn and develop age-appropriate toileting skills, however, age-related criteria in many areas for referral, means that opportunities for early intervention may be missed.

Strengths of an online survey methodology included the potential to reach a large, geographically and demographically diverse sample, at low cost and ease for participants, as demonstrated in other studies (24,25). Our surveys were developed and piloted with a diverse range of relevant stakeholders, parent carers and young people with neurodisability, ensuring that questions were relevant. Limitations included: the registration process led to potential delays and risk of participants entering contact details incorrectly; partial response, perhaps due to the length of the survey; responses were limited to those willing and able to use an online method; data presented are from across England, which may not adequately reflect variation in service provision and experience by locality, and can therefore only provide a snapshot of current practice; surveys were completed prior to the COVID-19 pandemic, and findings may be different when restrictions placed on health services during the pandemic are considered.

The survey findings highlighted perceived ability of the child and lack of adequate facilities as key factors affecting implementation of toileting interventions for CYP with neurodisability.

Confidential: For Review Only

This emphasises the requirement of proactive and timely individual assessments of need, to facilitate the introduction of an appropriate individualised toileting skills development programme, to give CYP the opportunity to become toilet trained or at least clean and dry at the same age as their typically developing peers. Previous guidance and research have also highlighted this need (23,26). The impact of incontinence on quality of life has previously been highlighted (23,27) and our findings reinforce the need for further research around availability and acceptability of interventions to adequately support CYP with neurodisability. Effectiveness of interventions is likely to impact opinions of acceptability; whilst the surveys gathered some information on perceived effectiveness (10), further evidence would add to the knowledge around appropriate incontinence support for CYP with neurodisability.

Confidential: For Review Only

Funding statement

This project was funded by the National Institute for Health and Care Research (NIHR) Health Technology Assessment programme (Award ID: 17/20/02). Helen Eke, Susan Ball, Stuart Logan, Rob Anderson, Harriet Hunt, Jo Thompson Coon, Rebecca Whear and Christopher Morris have previously been or are currently funded or supported by the NIHR Applied Research Collaboration South West Peninsula. The views expressed are those of the authors and not necessarily those of the NIHR or the Department of Health and Social Care.

Competing interests

The authors Helen Eke, Susan Ball, Annette Allinson, Rob Anderson, Harriet Hunt, Eve Hutton, Claire Lindsay, Stuart Logan, Julia Melluish, June Rogers, Jo Thompson Coon, Rebecca Whear, and Christopher Morris declare no competing interests.

Anne Wright is an Educational Chairperson of the International Children's Continence Society (not reimbursed), President of the charity ERIC (not reimbursed), and participates in the advisory board for Astellas DSMB Mirabegron.

Davina Richardson is employed by the charity Disabled Living, which receives unrestricted funding grants from the various commercial organisations to support the work of the charity. She is paediatric advisor to the Executive of the Association for Continence Advice, and is co-vice chair of the Paediatric Continence Forum (PCF), which is an independent multidisciplinary group of healthcare professionals campaigning for better care of bladder and bowel problems in children and young people, with a particular focus on community care.

Nick Madden is Honorary Secretary for Sutton and District Medical Society, and was previously vice-chair for the PCF.

Author contributions

HE and SB led the development of the surveys, analyses of the survey data and equally contributed to drafting the manuscript. All other authors were coinvestigators on the project, involved in the development of the surveys, and reviewed and approved the manuscript.

Data sharing

The full report and set of survey results is available from Open Research Exeter (ORE), DOI: https://doi.org/10.3310/hta25730.

Confidential: For Review Only

References [27/30]

1. Morris C, Janssens A, Tomlinson R, Williams J, Logan S. Towards a definition of neurodisability: A Delphi survey. Dev Med Child Neurol. 2013 Dec;55(12):1103–8.
2. Järvelin MR. Developmental history and neurological findings in enuretic children. Dev Med Child Neurol. 2008 Nov 12;31(6):728–36.
3. Foreman DM, Thambirajah MS. Conduct disorder, enuresis and specific developmental delays in two types of encopresis: a case-note study of 63 boys. Eur Child Adolesc Psychiatry. 1996 Apr;5(1):33–7.
4. Wendt L, Similä S, Niskanen P, Järvelin MR. Development of bowel and bladder control in the mentally retarded. Dev Med Child Neurol. 2010 Aug 24;32(6):515–8.
5. von Gontard A, de Jong TPVM, Rantell A, Nieuwhof-Leppink A, Badawi JK, Cardozo L. Do we manage incontinence in children and adults with special needs adequately? ICI-RS 2014. Neurology and Urodynamics. 2016 Feb;35(2):304–6.
6. Townsend J, Heng L, Thomas T, Egan M, Meade T. Costs of incontinence to families with severely handicapped children. J Public Health (Bangkok). 1981;3(2):119–22.
7. Von Gontard A, Nevéus T. The management of disorders of bladder and bowel control in childhood. Mac Keith Press; 2006.
8. Klassen TP, Kiddoo D, Lang ME, Friesen C, Russell K, Spooner C, et al. The effectiveness of different methods of toilet training for bowel and bladder control. Evidence Report / Technology Assessment (Full Report). 2006 Dec;(147):1–57.
9. Department for Education, Department of Health. Special educational needs and disability code of practice: 0 to 25 years. Statutory guidance for organisations which work with and support children and young people who have special educational needs or disabilities [Internet]. 2015 [cited 2022 Jun 14]. Available from: https://www.gov.uk/government/publications/send-code-of-practice-0-to-25
10. Eke H, Hunt H, Ball S, Rogers M, Whear R, Allinson A, et al. Improving continence in children and young people with neurodisability: a systematic review and survey. Health Technol Assess (Rockv). 2021 Nov;25(73):1–258.
11. Central University Research Ethics Committee (CUREC). Internet-Mediated Research [Internet]. 2021 Feb. Available from: http://ukrio.org/wp-content/uploads/UKRIO-Guidance-Note-Internet-Mediated-Research-v1.0.pdf
12. R Core Team. R: A Language and Environment for Statistical Computing [Internet]. Vienna, Austria: R Foundation for Statistical Computing; 2019. Available from: https://www.R-project.org/
13. Eysenbach G. Improving the quality of Web surveys: the Checklist for Reporting Results of Internet E-Surveys (CHERRIES). J Med Internet Res. 2004 Sep 29;6(3):e34.
14. Changing Places Consortium. What are Changing Places toilets? [Internet]. 2022 [cited 2022 Jun 14]. Available from: https://www.changing-places.org/
15. Weigel-Garrey C, Cook C, Brotherson M. Children and Privacy. Choice, Control, and Access in Home Environments. J Fam Issues. 1998;19(1):43–64.
16. Bauer SB, Saunders RA, Masoom SN, Choung K, Hayes LC, Price DE, et al. The art of introducing clean intermittent catheterization: How families respond and adapt: A qualitative study. Neurourol Urodyn. 2023 Jan 9;42(1):309–21.
17. Brazzelli M, Griffiths P V, Cody JD, Tappin D. Behavioural and cognitive interventions with or without other treatments for the management of faecal incontinence in children. Cochrane Database Syst Rev. 2011 Dec 7;(12).

Confidential: For Review Only

18. National Institute of Health and Care Excellence. Urinary incontinence in neurological disease: assessment and management Clinical guideline [Internet]. 2012. Available from: www.nice.org.uk/guidance/cg148
19. Murphy C, et al. Continence pad provision: meeting patients' fundamental care needs. Nursing Times (online) [Internet]. 2019 [cited 2022 Jun 14];115(2):39–42. Available from: https://www.nursingtimes.net/clinical-archive/continence/continence-pad-provision-meeting-patients-fundamental-care-needs-28-01-2019/
20. Richardson D. Guidance for the provision of continence containment products to children and young people. A consensus document. [Internet]. 2021 [cited 2022 Jun 14]. Available from: https://www.bbuk.org.uk/wp-content/uploads/2021/11/Guidance-for-the-provision-of-continence-containment-products-to-children-2021-2.pdf
21. Trajanovska M, Liew A, Gibb S, Goldfeld S, King SK. Retrospective audit of referral and triage pathways of paediatric patients with constipation and soiling. J Paediatr Child Health. 2020 Feb 1;56(2):298–303.
22. Eiselen C, Trajanovska M, Griffith A, Phan T, Goldfeld S, Gibb S, et al. Audit of enuresis referrals on the waiting list for a tertiary hospital outpatient clinic. J Paediatr Child Health. 2021 Oct 1;57(10):1645–50.
23. Kroeger KA, Sorensen-Burnworth R. Toilet training individuals with autism and other developmental disabilities: A critical review. Vol. 3, Research in Autism Spectrum Disorders. 2009. p. 607–18.
24. Lyons AC, Cude B, Lawrence FC, Gutter M. Conducting Research Online: Challenges Facing Researchers in Family and Consumer Sciences. Fam Consum Sci Res J. 2005;33(4):341–56.
25. Solomon DJ. Conducting web-based surveys. Practical Assessment, Research, and Evaluation. 2000;7(19).
26. Kroeger K, Sorensen R. A parent training model for toilet training children with autism. Journal of Intellectual Disability Research. 2010 Jun;54(6):556–67.
27. Ranasinghe N, Devanarayana NM, Benninga MA, Van Dijk M, Rajindrajith S. Psychological maladjustment and quality of life in adolescents with constipation. Arch Dis Child. 2017 Mar 1;102(3):268–73.

Confidential: For Review Only

Survey questions included in this paper

Parent carers

- What difficulties have you found using methods to help with toileting at home?
- If you and your child have experienced any of the following methods to help with toileting, please indicate how easy you found it using them at home.
- Where you live, please indicate which toileting products or equipment are provided for families of children and young people with special educational needs and/or a disability.
- Where you live, how acceptable is the waiting time for families to get the equipment and products they require once an assessment has been completed?

Children and young people

- How do you feel about . . . (list of different interventions)
- How do you feel about using the toilet … (list of different environments)

Health professionals

- Where you work, please indicate which toileting products or equipment are provided for families of children and young people with special educational needs and/or a disability.
- In your opinion, how acceptable is the waiting time in your area for families to receive the toileting support or equipment/products they require?
- In your opinion, to what extent do local commissioning/funding arrangements influence the toileting support you offer for individual children and young people with special educational needs and/or a disability (e.g., would you recommend the use of continence pads as they are supplied free of charge for families locally)?
- Where you work, do you have a bladder and bowel protocol/pathway for children and young people with special educational needs and/or a disability?
- Where you work, are there any toileting interventions for children and young people with special educational needs and/or a disability that are not provided, which you know are provided elsewhere?
- In your opinion, what do you think is the main barrier to developing a dedicated bladder and bowel pathway or service?
- In your opinion, is the provision of continence pads for children and young people with special educational needs and/or a disability a barrier or enabler for achieving continence?

School and social care staff

- Where you work, what difficulties have you found in helping children and young people to use the toileting methods (e.g., alarms or frames) provided?
- Where you work, how easy is it for you to provide or use the following methods to help children and young people with toileting?
- Where you work, please indicate which toileting products or equipment are provided for families of children and young people with special educational needs and/or a disability.

Box 1: List of survey questions included in analyses

Confidential: For Review Only

Table 1: Characteristics of survey respondents. Values are n (%) in respondent group

Parent carers		Children and young people		Health professionals		School and social care staff	
**Characteristic**	**N = 579**	**Characteristic**	**N = 20**	**Characteristic**	**N = 193**	**Characteristic**	**N = 119**
Child's gender		***Gender***		***Gender***		***Gender***	
Male	396 (68.4)	Male	6 (30)	Male	16 (8.3)	Male	6 (5.0)
Female	181 (31.3)	Female	14 (70)	Female	158 (81.9)	Female	111 (93.3)
pnts	2 (0.3)	pnts	0 (0)	pnts	7 (3.6)	pnts	1 (0.8)
na	0 (0)	na	0 (0)	na	12 (6.2)	na	1 (0.8)
Region of England where live		***Region of England where live***		***Region of England where work***		***Region of England where work***	
North West	40 (6.9)	North West	3 (15)	North West	21 (10.9)	North West	10 (8.4)
North East	21 (3.6)	North East	1 (5)	North East	12 (6.2)	North East	4 (3.4)
Y & H	31 (5.4)	Y & H	2 (10)	Y & H	9 (4.7)	Y & H	5 (4.2)
East Midlands	32 (5.5)	East Midlands	0 (0)	East Midlands	9 (4.7)	East Midlands	2 (1.7)
West Midlands	53 (9.2)	West Midlands	0 (0)	West Midlands	7 (3.6)	West Midlands	3 (2.5)
East Anglia	26 (4.5)	East Anglia	1 (5)	East Anglia	9 (4.7)	East Anglia	2 (1.7)
South West	166 (28.7)	South West	4 (20)	South West	39 (20.2)	South West	60 (50.4)
South East	123 (21.2)	South East	4 (20)	South East	26 (13.5)	South East	22 (18.5)
London	48 (8.3)	London	1 (5)	London	38 (19.7)	London	6 (5.0)
Outside England	28 (4.8)	Outside England	4 (20)	Outside England	18 (9.3)	Outside England	2 (1.7)
na	11 (1.9)	na	0 (0)	na	5 (2.6)	na	3 (2.5)
Age of child (years)		***Age (years)***				***Job role***	
Under 5	68 (11.7)	Under 5	0 (0)			Manager	19 (16.0)
5-7	136 (23.5)	5-7	0 (0)			Assistant	28 (23.5)
8-11	153 (26.4)	8-11	1 (5)			Teacher	29 (24.4)
12-17	157 (27.1)	12-17	5 (25)			Enabler	5 (4.2)
18-25	64 (11.1)	18-25	11 (55)			Social worker	0 (0)
Over 25	0 (0.0)	Over 25	3 (15)			Therapist	1 (0.8)
na	1 (0.2)	na	0 (0)			Other	36 (30.3)
Ethnicity		***Ethnicity***				na	1 (0.8)
White	540 (93.3)	White	19 (95)			***Where work***	
Mixed	13 (2.2)	Mixed	0 (0)			Mainstream school	30 (25.2)
Asian	10 (1.7)	Asian	0 (0)			Special school	51 (42.9)
Black	8 (1.4)	Black	0 (0)			Hospice / Respite care	0 (0)
Other	6 (1.0)	Other	1 (5)			Short break facility	5 (4.2)
na	2 (0.3)	na	0 (0)			Community care	6 (5.0)
Relationship to child		***How completed survey***				Primary care	1 (0.8)

Confidential: For Review Only

Mother*	522 (90.2)	On my own	17 (85)	Secondary care	0 (0)
Father	25 (4.3)	Someone is helping me	2 (10)	Other	25 (21.0)
Parent**	15 (2.6)	Someone is doing it for me	1 (5)	na	1 (0.8)
Grandparent	10 (1.7)	na	0 (0)		
Carer	2 (0.3)				
na	5 (0.9)				

pnts = prefer not to say; na = not answered; Y & H = Yorkshire & Humberside; * = including foster, adoptive and step mother; ** = including foster parent

Confidential: For Review Only

Table 2: Parent carers' and school and social care staff's perspectives on what difficulties they have found in helping children and young people to use toileting methods. Note: respondents were asked to choose all response options that applied

Response option, n (% of respondents)	Parent carers: Non-spinal cord pathology N = 536		Parent carers: Spinal cord pathology N = 20		School and social care staff N = 115	
No difficulties experienced	34	(6)	4	(20)	16	(14)
Child/young person's knowledge and understanding of what is required	328	(61)	9	(45)	67	(58)
Parent carer knowledge and understanding of what is required	56	(10)	4	(20)	54	(47)
Parent carer ability and time to focus on toileting	164	(31)	6	(30)	70	(61)
Child/young person's willingness*	329	(61)	14	(70)	-	-
Child/young person's adherence to the intervention**	-	-	-	-	48	(42)
Parent carer and child's lack of interest / motivation to change	67	(13)	1	(5)	40	(35)
Not enough training in how to use the methods offered*	52	(10)	1	(5)	-	-
Delays in professional assessments	149	(28)	2	(10)	44	(38)
Access to appropriate help and support	233	(43)	7	(35)	38	(33)
Funding and/or resources for equipment and products	126	(24)	3	(15)	53	(46)
Lack of consistency in support in different environments e.g., facility at home but not at school	205	(38)	4	(20)	69	(60)
Other	47	(9)	1	(5)	5	(4)

* Response option included in parent carer survey only.

** Response option included in school and social care staff survey only.

Confidential: For Review Only

Table 3: Children and young people's perspectives on how they feel about toileting interventions and about using the toilet in different places

n (% of respondents)	Very happy		OK		A bit unhappy		Very unhappy		Don't know OR this has never happened to me	
Intervention										
Following a special water/food diet (N = 20)	2	(10)	5	(25)	7	(35)	2	(10)	4	(20)
Using an alarm or timer to remind me to wee/poo (N = 20)	0	(0)	6	(30)	1	(5)	2	(10)	11	(55)
Using a hoist or a frame to help me use the toilet (N = 20)	1	(5)	7	(35)	1	(5)	1	(5)	10	(50)
Using pads, nappies or pull ups (N = 20)	3	(15)	3	(15)	1	(5)	5	(25)	8	(40)
Taking medication to help me wee/poo (N = 20)	4	(20)	2	(10)	5	(25)	1	(5)	8	(40)
Using a catheter or bowel washout (tubes that help you to wee or poo) (N = 20)	1	(5)	2	(10)	0	(0)	5	(25)	12	(60)
Having surgery (N = 20)	2	(10)	0	(0)	2	(10)	6	(30)	10	(50)
Place										
Using the toilet at home (N = 19)	12	(63)	2	(11)	3	(16)	1	(5)	1	(5)
Using the toilet at school / college / work (N = 20)	5	(25)	5	(25)	3	(15)	5	(25)	2	(10)
Using the toilet when out and about e.g., in a restaurant (N = 20)	2	(10)	3	(15)	9	(45)	5	(25)	1	(5)

Numbers are n (%) of respondents who gave an answer to the statement, so percentages add up to 100 across the rows.

Confidential: For Review Only

Table 4: Parent carers' and school and social care staff's perspectives on how easy specified toileting methods are to use at home, and how easy it is for them to provide or use specified methods to help children and young people with toileting, respectively

**Survey respondents / clinical group**	**Method, n (% of respondents)**	**How easy it is to use the method**									
		**Very easy**		**Easy**		**Difficult**		**Very difficult**		**Never used**	
**Parent carers / non-spinal cord pathology**	Dietary advice (N = 476) *(N = 244)*	37	(8) *(15)*	104	(22) *(43)*	64	(13) *(26)*	39	(8) *(16)*	232	(49)
	Fluid intake advice (N = 491) *(N = 324)*	51	(10) *(16)*	140	(29) *(43)*	101	(21) *(31)*	32	(7) *(10)*	167	(34)
	Behavioural intervention e.g., reward chart, timer, alarm (N = 483) *(N = 292)*	18	(4) *(6)*	72	(15) *(25)*	99	(20) *(34)*	103	(21) *(35)*	191	(40)
	Simple aid/equipment e.g., raised step or seat (N = 479) *(N = 292)*	60	(13) *(21)*	156	(33) *(53)*	54	(11) *(18)*	22	(5) *(8)*	187	(39)
	Bespoke aid/equipment e.g., hoist or frame (N = 456) *(N = 77)*	12	(3) *(16)*	31	(7) *(40)*	22	(5) *(29)*	12	(3) *(16)*	379	(83)
	Housing adaptions e.g., specialised toilet (N = 456) *(N = 68)*	16	(4) *(24)*	23	(5) *(34)*	15	(3) *(22)*	14	(3) *(21)*	388	(85)
	Continence products e.g., nappies, pads, pull ups (N = 497) *(N = 401)*	132	(27) *(33)*	188	(38) *(47)*	60	(12) *(15)*	21	(4) *(5)*	96	(19)
	Medication e.g., laxatives, anticholinergics, desmopressin (N = 489) *(N = 295)*	55	(11) *(19)*	131	(27) *(44)*	73	(15) *(25)*	36	(7) *(12)*	194	(40)
	Catheters (N = 442) *(N = 14)*	2	(<1) *(14)*	3	(<1) *(21)*	5	(1) *(36)*	4	(1) *(29)*	428	(97)
	Colonic enema (bowel washout) (N = 450) *(N = 29)*	4	(1) *(14)*	11	(2) *(38)*	3	(<1) *(10)*	11	(2) *(38)*	421	(94)

Confidential: For Review Only

	Surgical intervention e.g., botox, bladder reconstruction, mitrofanoff (N = 447) *(N = 19)*	1	(<1) *(5)*	6	(1) *(32)*	4	(1) *(21)*	8	(2) *(42)*	428	(96)
		**Very easy**		**Easy**		**Difficult**		**Very difficult**		**Never used**	
**Parent carers / spinal cord pathology**	Dietary advice (N = 18) *(N = 11)*	5	(28) *(45)*	2	(11) *(18)*	4	(22) *(36)*	0	(0) *(0)*	7	(39)
	Fluid intake advice (N = 18) *(N = 13)*	4	(22) *(31)*	3	(17) *(23)*	5	(28) *(38)*	1	(6) *(8)*	5	(28)
	Behavioural intervention e.g., reward chart, timer, alarm (N = 17) *(N = 7)*	0	(0) *(0)*	3	(18) *(43)*	2	(12) *(29)*	2	(12) *(29)*	10	(59)
	Simple aid/equipment e.g., raised step or seat (N = 20) *(N = 12)*	5	(25) *(42)*	3	(15) *(25)*	2	(10) *(17)*	2	(10) *(17)*	8	(40)
	Bespoke aid/equipment e.g., hoist or frame (N = 18) *(N = 4)*	1	(6) *(25)*	1	(6) *(25)*	2	(11) *(50)*	0	(0) *(0)*	14	(78)
	Housing adaptions e.g., specialised toilet (N = 19) *(N = 5)*	2	(11) *(40)*	3	(16) *(60)*	0	(0) *(0)*	0	(0) *(0)*	14	(74)
	Continence products e.g., nappies, pads, pull ups (N = 19) *(N = 16)*	7	(37) *(44)*	8	(42) *(50)*	1	(5) *(6)*	0	(0) *(0)*	3	(16)
	Medication e.g., laxatives, anticholinergics, desmopressin (N = 18) *(N = 17)*	6	(33) *(35)*	4	(22) *(24)*	4	(22) *(24)*	3	(17) *(18)*	1	(6)
	Catheters (N = 19) *(N = 15)*	4	(21) *(27)*	8	(42) *(53)*	2	(11) *(13)*	1	(5) *(7)*	4	(21)
	Colonic enema (bowel washout) (N = 18) *(N = 11)*	1	(6) *(9)*	6	(33) *(55)*	1	(6) *(9)*	3	(17) *(27)*	7	(39)
	Surgical intervention e.g., botox, bladder reconstruction, mitrofanoff (N = 18) *(N = 6)*	2	(11) *(33)*	2	(11) *(33)*	1	(6) *(17)*	1	(6) *(17)*	12	(67)

Confidential: For Review Only

		Very easy		Easy		Difficult		Very difficult		Never used	
**School and social care staff / not separated by clinical group**	Dietary advice (N = 116) *(N = 96)*	17	(15) *(18)*	59	(51) *(61)*	19	(16) *(20)*	1	(1) *(1)*	20	(17)
	Fluid intake advice (N = 116) *(N = 106)*	24	(21) *(23)*	64	(55) *(60)*	16	(14) *(15)*	2	(2) *(2)*	10	(9)
	Behavioural intervention e.g., reward charts, timers, alarms (N = 115) *(N = 109)*	46	(40) *(42)*	52	(45) *(48)*	10	(9) *(9)*	1	(1) *(1)*	6	(5)
	Physical aids / equipment e.g., specialised toilets, hoists, frames (N = 116) *(N = 99)*	30	(26) *(30)*	47	(41) *(47)*	15	(13) *(15)*	7	(6) *(7)*	17	(15)
	Continence products e.g., nappies, pads, pull ups, continence pants (N = 114) *(N = 102)*	34	(30) *(33)*	49	(43) *(48)*	15	(13) *(15)*	4	(4) *(4)*	12	(11)
	Medication or drugs e.g., laxatives, anticholinergics, desmopressin (N = 113) *(N = 72)*	15	(13) *(21)*	37	(33) *(51)*	19	(17) *(26)*	1	(1) *(1)*	41	(36)
	Catheterisation (N = 113) *(N = 31)*	8	(7) *(26)*	11	(10) *(35)*	9	(8) *(29)*	3	(3) *(10)*	82	(73)
	Colonic enema (bowel washout) (N = 112) *(N = 21)*	1	(1) *(5)*	5	(4) *(24)*	12	(11) *(57)*	3	(3) *(14)*	91	(81)
	Surgical intervention e.g., cytoscopic botox, ACE, bladder reconstruction, mitrofanoff (N = 109) *(N = 16)*	2	(2) *(13)*	4	(4) *(25)*	8	(7) *(50)*	2	(2) *(13)*	93	(85)

Numbers in italic show the total number of respondents (*N*) and percentage of respondents choosing the response option, excluding those who answered ‘Never used’. ACE = antegrade colonic enema.

Confidential: For Review Only

Confidential: For Review Only

Table 5: Health professionals', school and social care staff's, and parent carers' perspectives on provision of interventions to families in their area

Response option, n (% of respondent group)	Health professionals												School and social care staff		Parent carers			
	BBS Nurses		Nurses		Paediatricians		Surgeons		Therapists		Other				Non-spinal cord pathology		Spinal cord pathology	
**Behavioural - e.g., timer, alarm**	**N = 20**		**N = 74**		**N = 29**		**N = 15**		**N = 27**		**N = 12**		**N = 112**		**N = 519**		**N = 17**	
Free of charge	12	(60)	26	(35)	14	(48)	5	(33)	5	(19)	4	(33)	22	(20)	29	(6)	0	(0)
Subsidised	0	(0)	0	(0)	2	(7)	0	(0)	1	(4)	1	(8)	3	(3)	1	(<1)	0	(0)
Available to purchase	0	(0)	10	(14)	0	(0)	1	(7)	4	(15)	0	(0)	18	(16)	46	(9)	0	(0)
Unavailable	6	(30)	26	(35)	4	(14)	6	(40)	11	(41)	6	(50)	14	(13)	49	(9)	2	(12)
Don't know (/ never used)**	2	(10)	12	(16)	9	(31)	3	(20)	6	(22)	1	(8)	55	(49)	394	(76)	15	(88)
**Simple aid/equipment - e.g., raised seat or step**	**N = 18**		**N = 74**		**N = 29**		**N = 15**		**N = 27**		**N = 12**		**N = 113***		**N = 523**		**N = 19**	
Free of charge	7	(39)	27	(36)	11	(38)	2	(13)	20	(74)	3	(25)	43	(38)	91	(17)	8	(42)
Subsidised	1	(6)	1	(1)	0	(0)	1	(7)	0	(0)	0	(0)	7	(6)	2	(<1)	0	(0)
Available to purchase	2	(11)	12	(16)	7	(24)	1	(7)	1	(4)	1	(8)	18	(16)	69	(13)	2	(11)
Unavailable	5	(28)	25	(34)	3	(10)	6	(40)	3	(11)	5	(42)	13	(12)	45	(9)	2	(11)
Don't know (/ never used)**	3	(17)	9	(12)	8	(28)	5	(33)	3	(11)	3	(25)	32	(28)	316	(60)	7	(37)
**Bespoke aid/equipment - e.g., hoist or frame**	**N = 18**		**N = 74**		**N = 29**		**N = 15**		**N = 27**		**N = 12**				**N = 521**		**N = 17**	
Free of charge	9	(50)	42	(57)	18	(62)	0	(0)	20	(74)	2	(17)			73	(14)	2	(12)

Subsidised	2	(11)	2	(3)	0	(0)	1	(7)	1	(4)	1	(8)			5	(1)	0	(0)
Available to purchase	1	(6)	3	(4)	0	(0)	0	(0)	2	(7)	0	(0)			12	(2)	0	(0)
Unavailable	3	(17)	15	(20)	1	(3)	6	(40)	1	(4)	4	(33)			20	(4)	0	(0)
Don't know (/ never used)**	3	(17)	12	(16)	10	(34)	8	(53)	3	(11)	5	(42)			411	(79)	15	(88)
**Housing adaption - e.g., specialised toilet**	**N = 18**		**N = 72**		**N = 29**		**N = 15**		**N = 27**		**N = 12**		**N = 113**		**N = 516**		**N = 19**	
Free of charge	7	(39)	26	(36)	15	(52)	0	(0)	13	(48)	3	(25)	29	(26)	61	(12)	5	(26)
Subsidised	3	(17)	5	(7)	3	(10)	1	(7)	4	(15)	1	(8)	14	(12)	19	(4)	1	(5)
Available to purchase	1	(6)	4	(6)	0	(0)	0	(0)	2	(7)	0	(0)	9	(8)	14	(3)	1	(5)
Unavailable	3	(17)	14	(19)	1	(3)	6	(40)	4	(15)	4	(33)	11	(10)	20	(4)	0	(0)
Don't know (/ never used)**	4	(22)	23	(32)	10	(34)	8	(53)	4	(15)	4	(33)	50	(44)	402	(78)	12	(63)
**Products - e.g., pads, nappies, continence pants, pull ups**	**N = 18**		**N = 72**		**N = 29**		**N = 14**		**N = 26**		**N = 12**		**N = 115**		**N = 534**		**N = 19**	
Free of charge	17	(94)	53	(74)	19	(66)	8	(57)	11	(42)	6	(50)	55	(48)	256	(48)	9	(47)
Subsidised	1	(6)	5	(7)	4	(14)	0	(0)	4	(15)	1	(8)	8	(7)	21	(4)	1	(5)
Available to purchase	0	(0)	2	(3)	1	(3)	0	(0)	0	(0)	0	(0)	17	(15)	55	(10)	6	(32)
Unavailable	0	(0)	7	(10)	0	(0)	3	(21)	2	(8)	4	(33)	13	(11)	42	(8)	1	(5)
Don't know (/ never used)**	0	(0)	5	(7)	5	(17)	3	(21)	9	(35)	1	(8)	22	(19)	160	(30)	2	(11)
**Medications - e.g., laxatives**	**N = 18**		**N = 72**		**N = 29**		**N = 14**		**N = 26**		**N = 12**		**N = 113**		**N = 522**		**N = 18**	
Free of charge	15	(83)	60	(83)	29	(100)	13	(93)	8	(31)	9	(75)	47	(42)	290	(56)	17	(94)
Subsidised	1	(6)	0	(0)	0	(0)	1	(7)	0	(0)	0	(0)	2	(2)	3	(<1)	0	(0)
Available to purchase	0	(0)	0	(0)	0	(0)	0	(0)	0	(0)	1	(8)	7	(6)	15	(3)	0	(0)

Confidential: For Review Only

Unavailable	1	(6)	10	(14)	0	(0)	0	(0)	2	(8)	2	(17)	15	(13)	6	(1)	0	(0)
Don't know (/ never used)**	1	(6)	2	(3)	0	(0)	0	(0)	16	(62)	0	(0)	42	(37)	208	(40)	1	(6)

* = Aids/equipment not separated into simple or bespoke in school and social care survey; ** = response option includes *never used* for school and social care staff and parent carer surveys; BBS = Bladder and Bowel Specialist.

Confidential: For Review Only

Table 6: Parent carers' and health professionals' perspectives on the acceptability of waiting times for families to receive the required toileting support or equipment/products

	Parent carers				Health professionals											
**Response option, n (% of respondent group)**	**Non-spinal cord pathology**		**Spinal cord pathology**		**BBS Nurses**		**Nurses**		**Paediatricians**		**Surgeons**		**Therapists**		**Other**	
	**N = 553**		**N = 20**		**N = 22**		**N = 79**		**N = 30**		**N = 15**		**N = 33**		**N = 13**	
Very acceptable	27	(5)	3	(15)	4	(18)	5	(6)	0	(0)	2	(13)	2	(6)	0	(0)
Acceptable	121	(22)	7	(35)	8	(36)	32	(41)	11	(37)	3	(20)	9	(27)	7	(54)
Unacceptable	79	(14)	3	(15)	7	(32)	17	(22)	9	(30)	7	(47)	11	(33)	4	(31)
Very unacceptable	64	(12)	2	(10)	2	(9)	9	(11)	2	(7)	1	(7)	1	(3)	1	(8)
Don't know	262	(47)	5	(25)	1	(5)	16	(20)	8	(27)	2	(13)	10	(30)	1	(8)

BBS = Bladder and Bowel Specialist.

Confidential: For Review Only

Confidential: For Review Only

Table 7: Health professionals' perspectives on: (a) the extent to which local commissioning/funding arrangements influence the toileting support they offer for children and young people with neurodisability; (b) whether, where they work, there is a bladder and bowel protocol/pathway for children and young people with neurodisability; (c) whether there are any toileting interventions for children and young people with neurodisability that are not provided, which they know are provided elsewhere; (d) what they think is the main barrier to developing a dedicated bladder and bowel pathway or service. Responses to (d) are shown for those health professionals who answered either 'yes' or 'no' to the question of whether there is a bladder and bowel protocol/pathway for children and young people with neurodisability - those who answered 'don't know' are excluded

**Question**	**Response option, n (% of respondents in role)**	**BBS Nurses**		**Nurses**		**Paediatricians**		**Surgeons**		**Therapists**		**Other**	
		**N = 22**		**N = 79**		**N = 31**		**N = 15**		**N = 33**		**N = 13**	
(a) *To what extent do local commissioning/funding arrangements influence the toileting support that you offer?*	Never	5	(23)	12	(15)	2	(6)	2	(13)	6	(18)	1	(8)
	Sometimes	6	(27)	18	(23)	10	(32)	5	(33)	8	(24)	5	(38)
	Often	3	(14)	11	(14)	10	(32)	4	(27)	5	(15)	1	(8)
	Always	6	(27)	19	(24)	6	(19)	1	(7)	1	(3)	1	(8)
	Don't know	2	(9)	19	(24)	3	(10)	3	(20)	13	(39)	5	(38)
		**N = 22**		**N = 79**		**N = 30**		**N = 15**		**N = 33**		**N = 13**	
(b) *Is there a bladder and bowel protocol/pathway?*	Yes	17	(77)	46	(58)	10	(33)	10	(67)	5	(15)	5	(38)
	No	4	(18)	21	(27)	17	(57)	4	(27)	13	(39)	4	(31)
	Don't know	1	(5)	12	(15)	3	(10)	1	(7)	15	(45)	4	(31)

(c) are there any toileting interventions that are not provided, which they know are provided elsewhere?		**N = 22**		**N = 79**		**N = 30**		**N = 15**		**N = 33**		**N = 13**	
	Yes	6	(27)	18	(23)	8	(27)	6	(27)	18	(23)	8	(27)
	No	11	(50)	26	(33)	4	(13)	11	(50)	26	(33)	4	(13)
	Don't know	5	(23)	35	(44)	18	(60)	5	(23)	35	(44)	18	(60)
(d) what is the main barrier to developing a dedicated bladder and bowel pathway or service		**N = 21**		**N = 67**		**N = 27**		**N = 14**		**N = 18**		**N = 9**	
	Lack of funding and/or resources	16	(76)	38	(57)	17	(63)	16	(76)	38	(57)	17	(63)
	Lack of professional interest	1	(5)	9	(13)	2	(7)	1	(5)	9	(13)	2	(7)
	Time	0	(0)	10	(15)	3	(11)	0	(0)	10	(15)	3	(11)
	Lack of need in local area	0	(0)	1	(1)	2	(7)	0	(0)	1	(1)	2	(7)
	Don't know	1	(5)	6	(9)	1	(4)	1	(5)	6	(9)	1	(4)
	Other	3	(14)	3	(4)	2	(7)	3	(14)	3	(4)	2	(7)

BBS = Bladder and Bowel Specialist.

Confidential: For Review Only

Table 8: Health professionals' perspectives on whether provision of continence pads for children and young people with special educational needs and/or a disability is a barrier or enabler for achieving continence

**Response option, n (% of respondents in role)**	**BBS Nurses**		**Nurses**		**Paediatricians**		**Surgeons**		**Therapists**		**Other**	
	**N = 22**		**N = 76**		**N = 30**		**N = 15**		**N = 33**		**N = 13**	
Barrier	16	(73)	39	(51)	7	(23)	1	(7)	9	(27)	5	(38)
Enabler	4	(18)	18	(24)	10	(33)	6	(40)	7	(21)	5	(38)
Don't know	2	(9)	19	(25)	13	(43)	8	(53)	17	(52)	3	(23)

BBS = Bladder and Bowel Specialist.

Confidential: For Review Only

Recruiting now!

We are conducting an online survey about toileting with health professionals, parent/carers, young people and education and social care staff.

The NHS wants to improve toileting services for children and young people with special educational needs and disability aged up to 25 years. We need to gather the views and experiences of families, clinicians, and school and social care staff, to establish how toileting is managed currently. The results of the survey will help inform health and care services for children in the future.

For further information and to take part in the survey, visit:
http://sites.exeter.ac.uk/iconstudy/taking-part/

ICoN improving continence for children and young people with neurodisability

FUNDED BY NIHR | National Institute for Health Research

For Review Only

Checklist for Reporting Results of Internet E-Surveys (CHERRIES)

Checklist Item	***Explanation***	***Page Number***
Describe survey design	Describe target population, sample frame. Is the sample a convenience sample? (In "open" surveys this is most likely.)	6
IRB approval	Mention whether the study has been approved by an IRB.	7
Informed consent	Describe the informed consent process. Where were the participants told the length of time of the survey, which data were stored and where and for how long, who the investigator was, and the purpose of the study?	7
Data protection	If any personal information was collected or stored, describe what mechanisms were used to protect unauthorized access.	7
Development and testing	State how the survey was developed, including whether the usability and technical functionality of the electronic questionnaire had been tested before fielding the questionnaire.	6
Open survey versus closed survey	An "open survey" is a survey open for each visitor of a site, while a closed survey is only open to a sample which the investigator knows (password-protected survey).	6
Contact mode	Indicate whether or not the initial contact with the potential participants was made on the Internet. (Investigators may also send out questionnaires by mail and allow for Web-based data entry.)	6
Advertising the survey	How/where was the survey announced or advertised? Some examples are offline media (newspapers), or online (mailing lists – If yes, which ones?) or banner ads (Where were these banner ads posted and what did they look like?). It is important to know the wording of the announcement as it will heavily influence who chooses to participate. Ideally the survey announcement should be published as an appendix.	6
Web/E-mail	State the type of e-survey (eg, one posted on a Web site, or one sent out through e-mail). If it is an e-mail survey, were the responses entered manually into a database, or was there an automatic method for capturing responses?	6
Context	Describe the Web site (for mailing list/newsgroup) in which the survey was posted. What is the Web site about, who is visiting it, what are visitors normally looking for? Discuss to what degree the content of the Web site could pre-select the sample or influence the results. For example, a survey about vaccination on a anti-immunization Web site will have different results from a Web survey conducted on a government Web site	6
Mandatory/voluntary	Was it a mandatory survey to be filled in by every visitor who wanted to enter the Web site, or was it a voluntary survey?	6
Incentives	Were any incentives offered (eg, monetary, prizes, or non-monetary incentives such as an offer to provide the survey results)?	6

Confidential: For Review Only

Time/Date	In what timeframe were the data collected?	7
Randomization of items or questionnaires	To prevent biases items can be randomized or alternated.	6
Adaptive questioning	Use adaptive questioning (certain items, or only conditionally displayed based on responses to other items) to reduce number and complexity of the questions.	6
Number of Items	What was the number of questionnaire items per page? The number of items is an important factor for the completion rate.	6
Number of screens (pages)	Over how many pages was the questionnaire distributed? The number of items is an important factor for the completion rate.	6
Completeness check	It is technically possible to do consistency or completeness checks before the questionnaire is submitted. Was this done, and if “yes”, how (usually JAVAScript)? An alternative is to check for completeness after the questionnaire has been submitted (and highlight mandatory items). If this has been done, it should be reported. All items should provide a non-response option such as “not applicable” or “rather not say”, and selection of one response option should be enforced.	7
Review step	State whether respondents were able to review and change their answers (eg, through a Back button or a Review step which displays a summary of the responses and asks the respondents if they are correct).	7
Unique site visitor	If you provide view rates or participation rates, you need to define how you determined a unique visitor. There are different techniques available, based on IP addresses or cookies or both.	N/A
View rate (Ratio of unique survey visitors/unique site visitors)	Requires counting unique visitors to the first page of the survey, divided by the number of unique site visitors (not page views!). It is not unusual to have view rates of less than 0.1 % if the survey is voluntary.	N/A
Participation rate (Ratio of unique visitors who agreed to participate/unique first survey page visitors)	Count the unique number of people who filled in the first survey page (or agreed to participate, for example by checking a checkbox), divided by visitors who visit the first page of the survey (or the informed consents page, if present). This can also be called “recruitment” rate.	8
Completion rate (Ratio of users who finished the survey/users who	The number of people submitting the last questionnaire page, divided by the number of people who agreed to participate (or submitted the first survey page). This is only relevant if there is a separate “informed consent” page or if the survey goes over several pages. This is a measure for attrition. Note that	8

Confidential: For Review Only

agreed to participate)	"completion" can involve leaving questionnaire items blank. This is not a measure for how completely questionnaires were filled in. (If you need a measure for this, use the word "completeness rate".)	
Cookies used	Indicate whether cookies were used to assign a unique user identifier to each client computer. If so, mention the page on which the cookie was set and read, and how long the cookie was valid. Were duplicate entries avoided by preventing users access to the survey twice; or were duplicate database entries having the same user ID eliminated before analysis? In the latter case, which entries were kept for analysis (eg, the first entry or the most recent)?	7
IP check	Indicate whether the IP address of the client computer was used to identify potential duplicate entries from the same user. If so, mention the period of time for which no two entries from the same IP address were allowed (eg, 24 hours). Were duplicate entries avoided by preventing users with the same IP address access to the survey twice; or were duplicate database entries having the same IP address within a given period of time eliminated before analysis? If the latter, which entries were kept for analysis (eg, the first entry or the most recent)?	7
Log file analysis	Indicate whether other techniques to analyze the log file for identification of multiple entries were used. If so, please describe.	7
Registration	In "closed" (non-open) surveys, users need to login first and it is easier to prevent duplicate entries from the same user. Describe how this was done. For example, was the survey never displayed a second time once the user had filled it in, or was the username stored together with the survey results and later eliminated? If the latter, which entries were kept for analysis (eg, the first entry or the most recent)?	6
Handling of incomplete questionnaires	Were only completed questionnaires analyzed? Were questionnaires which terminated early (where, for example, users did not go through all questionnaire pages) also analyzed?	7
Questionnaires submitted with an atypical timestamp	Some investigators may measure the time people needed to fill in a questionnaire and exclude questionnaires that were submitted too soon. Specify the timeframe that was used as a cut-off point, and describe how this point was determined.	7
Statistical correction	Indicate whether any methods such as weighting of items or propensity scores have been used to adjust for the non-representative sample; if so, please describe the methods.	7

Confidential: For Review Only

This checklist has been modified from Eysenbach G. Improving the quality of Web surveys: the Checklist for Reporting Results of Internet E-Surveys (CHERRIES). J Med Internet Res. 2004 Sep 29;6(3):e34 [erratum in J Med Internet Res. 2012; 14(1): e8.]. Article available at https://www.jmir.org/2004/3/e34/; erratum available https://www.jmir.org/2012/1/e8/. Copyright ©Gunther Eysenbach. Originally published in the Journal of Medical Internet Research, 29.9.2004 and 04.01.2012.

This is an open-access article distributed under the terms of the Creative Commons Attribution License (https://creativecommons.org/licenses/by/2.0/), which permits unrestricted use, distribution, and reproduction in any medium, provided the original work, first published in the Journal of Medical Internet Research, is properly cited.

Confidential: For Review Only

BMJ Paediatrics Open

Interventions to improve continence for children and young people with neurodisability: a national survey of practitioner and family perspectives and experiences

Journal:	*BMJ Paediatrics Open*
Manuscript ID	bmjpo-2023-002238.R1
Article Type:	Original research
Date Submitted by the Author:	01-Nov-2023
Complete List of Authors:	Eke, Helen; University of Exeter, Medical School<br>Ball, Susan; University of Exeter, Medical School<br>Allinson, Annette; University of Exeter, Peninsula Childhood Disability Research Unit<br>Anderson, Rob; University of Exeter, Medical School<br>Hunt, Harriet; University of Exeter, Medical School<br>Hutton, Eve; Canterbury Christ Church University<br>Lindsay, Claire; Northern Devon Healthcare NHS Trust<br>Logan, Stuart; University of Exeter, Child Health, Medical School<br>Madden, Nicholas; Chelsea and Westminster Hospital NHS Foundation Trust<br>Melluish, Julia; University of Exeter, Peninsula Childhood Disability Research Unit<br>Richardson, Davina; Disabled Living Foundation<br>Rogers, June; Disabled Living Foundation<br>Thompson Coon, Jo; University of Exeter, National Institute for Health Research (NIHR) Applied Research Collaboration (ARC) South West Peninsula (PenARC)<br>Whear, Rebecca; University of Exeter, National Institute for Health Research (NIHR) Applied Research Collaboration (ARC) South West Peninsula (PenARC)<br>Wright, Anne; Guy's and St Thomas' Hospitals NHS Trust<br>Morris, Christopher; University of Exeter, Child Health, Medical School
Keywords:	Neurodisability, Enuresis, Health services research

SCHOLARONE™
Manuscripts

I, the Submitting Author has the right to grant and does grant on behalf of all authors of the Work (as defined in the below author licence), an exclusive licence and/or a non-exclusive licence for contributions from authors who are: i) UK Crown employees; ii) where BMJ has agreed a CC-BY licence shall apply, and/or iii) in accordance with the terms applicable for US Federal Government officers or employees acting as part of their official duties; on a worldwide, perpetual, irrevocable, royalty-free basis to BMJ Publishing Group Ltd ("BMJ") its licensees and where the relevant Journal is co-owned by BMJ to the co-owners of the Journal, to publish the Work in this journal and any other BMJ products and to exploit all rights, as set out in our licence.

The Submitting Author accepts and understands that any supply made under these terms is made by BMJ to the Submitting Author unless you are acting as an employee on behalf of your employer or a postgraduate student of an affiliated institution which is paying any applicable article publishing charge ("APC") for Open Access articles. Where the Submitting Author wishes to make the Work available on an Open Access basis (and intends to pay the relevant APC), the terms of reuse of such Open Access shall be governed by a Creative Commons licence – details of these licences and which Creative Commons licence will apply to this Work are set out in our licence referred to above.

Other than as permitted in any relevant BMJ Author's Self Archiving Policies, I confirm this Work has not been accepted for publication elsewhere, is not being considered for publication elsewhere and does not duplicate material already published. I confirm all authors consent to publication of this Work and authorise the granting of this licence.

Confidential: For Review Only

Interventions to improve continence for children and young people with neurodisability: a national survey of practitioner and family perspectives and experiences

Eke Helen[1,2*†], Ball Susan[1*], Allinson Annette[2], Anderson Rob[3], Hunt Harriet[3], Hutton Eve[4], Lindsay Claire[5], Logan Stuart[1,2], Madden Nicholas[6], Melluish Julia[2], Richardson Davina[7], Rogers June[7], Thompson Coon Jo[1,3], Whear Rebecca[1,3], Wright Anne[8], Morris Christopher[1,2]

*Equal contribution; †Corresponding author h.e.eke@exeter.ac.uk ORCID 0000-0003-4781-6683

[1] National Institute for Health Research (NIHR) Applied Research Collaboration (ARC) South West Peninsula (PenARC), Exeter, UK

[2] Peninsula Childhood Disability Research Unit (PenCRU), University of Exeter Medical School, University of Exeter, Exeter, UK

[3] Evidence Synthesis & Modelling for Health Improvement (ESMI), University of Exeter Medical School, University of Exeter, Exeter, UK

[4] School of Allied Health Professions, Canterbury Christ Church University, Canterbury, UK

[5] Paediatric Bladder and Bowel Care Team, Northern Devon Healthcare NHS Trust, Exeter, UK

[6] Children's Surgery, Chelsea and Westminster Hospital NHS Foundation Trust, London, UK

[7] Bladder & Bowel UK, Disabled Living, Manchester, UK

[8] Evelina London Children's Hospital, Guy's and St Thomas' NHS Foundation Trust, London, UK

Word count [text excluding refs 3436]

Confidential: For Review Only

Abstract [284/300 words]

Objective: Describe families' experiences of interventions to improve continence in children and young people with neurodisability, and health professionals' and school and social care staff's perspectives regarding factors affecting intervention use.

Design: Four online surveys were developed and advertised to parent carers, young people with neurodisability, health professionals and school and social care staff, via societies, charities, professional contacts, schools, local authorities, and national parent carer and family forums, who shared invitations with their networks. Survey questions explored: difficulties helping children and young people use interventions; acceptability of interventions and waiting times; ease of use and availability of interventions, and facilitators and barriers to improving continence.

Results: 1028 parent carers, 26 young people, 352 health professionals and 202 school and social care staff registered to participate. Completed surveys were received from 579 (56.3%) parent carers, 20 (77%) young people, 193 (54.8%) health professionals, and 119 (58.9%) school and social care staff. Common parent carer-reported difficulties in using interventions to help their children and young people to learn to use the toilet included their child's lack of understanding about what was required (reported by 337/556 (60.6%) parent carers who completed question) and their child's lack of willingness (343/556, 61.7%). Almost all (142/156, 91%) health professionals reported lack of funding and resources as barriers to provision of continence services. Many young people (14/19, 74%) were unhappy using toilet facilities whilst out and about.

Conclusions: Perceptions that children lack understanding and willingness, and inadequate facilities, impact the implementation of toileting interventions for children and young people with neurodisability. Greater understanding is needed for children to learn developmentally appropriate toileting skills. Further research is recommended around availability and acceptability of interventions to ensure quality of life is unaffected.

For Review Only

What is already known on this topic

- Children with neurodisability are often delayed in acquiring continence and are more frequently incontinent than typically developing children. Social, economic, and environmental factors, parenting strategies and child behaviour all affect toilet training.
- Incontinence can affect the quality of life of the child or young person and their carers.
- Many children with neurodisability can become continent, and, while not all children are able to become fully independent in toileting, many can improve their continence with support, with consequent positive gains in quality of life.
- A variety of approaches and interventions are available including information and support, charts to monitor/feedback, schedules for drinking and toileting, cognitive behavioural approaches, equipment, aids, relaxation techniques, psychotherapy, group-based programmes, medicines, and surgery. Previous research has identified limited evidence for toilet training strategies for children with physical and learning disabilities.

What this paper adds

- Families describe inadequate access to continence support and interventions.
- Many children are perceived as lacking willingness and understanding regarding continence needs.
- Many health professionals reported poor local availability of some interventions, and a lack of funding for dedicated continence services.
- Children and young people were unhappy with some interventions and with out-of-home toilet facilities.

How this study might affect research, practice or policy

- Highlights the need for further research around acceptability of interventions to improve continence in children and young people with neurodisability, without compromising quality of life.
- Emphasises the requirement for an individual assessment of needs for children and young people with neurodisability, to be carried out proactively and in a timely manner.
- Funding issues are likely restricting the toileting interventions that are being offered to children and young people with neurodisability.

Confidential: For Review Only

Introduction

Neurodisability describes a group of congenital or acquired long-term conditions attributed to the impairment of the brain and/or neuromuscular system, creating functional limitations including difficulties with movement, cognition, hearing, vision, communication, emotion, and behaviour (1). Children with neurodisability are often delayed in acquiring continence, and more frequently incontinent than typically developing children; they may be slower to learn to manage toileting or may need additional support (1–3). Social, economic, and environmental factors, parenting strategies and child behaviour can all affect toilet training.

Continence is being clean and dry without using containment products such as pads/nappies. Incontinence can affect the quality of life of the young person and their carers (4) and the long-term physical, psychological and financial burden can be considerable (5). The spectrum of neurodisability is vast and some children may never reach a physical or psychological developmental stage where continence is an achievable goal. Many children with neurodisability however can become continent, and, while not all are able to become fully independent in toileting, many can improve their continence with support (2–4), with consequent improvement in quality of life. Distinguishing continence for individuals with and without spinal cord pathology affecting bladder and bowel sensorimotor control is therefore crucial. Without sensation and motor control, and normal detrusor, colonic and sphincter function, assistive technology or alternative approaches to bladder and bowel storage and emptying will often be needed.

A variety of approaches and interventions are available (6) including information and support, monitoring/feedback charts, drinking and toileting schedules, cognitive behavioural approaches, equipment, aids, relaxation techniques, psychotherapy, group-based programmes, medicines and surgery (7). An existing systematic review identified limited evidence for toilet training strategies for children with physical and learning disabilities (8).

Research to evaluate ways to promote continence for children with neurodisability ranked 7/10 in the British Academy of Childhood Disability James Lind Alliance Research Priority Setting Partnership (9). Subsequently, the ICoN (Improving Continence in children and young people with Neurodisability) study was commissioned by the National Institute for Health and Care Research to summarise available evidence for interventions and practice relating to improving continence for children and young people with neurodisability (10). Online surveys were conducted with parent carers, children and young people with neurodisability, health

Confidential: For Review Only

professionals and school and social care staff. This paper details the survey process and aims to describe the experiences and perspectives of parent carers and children and young people with neurodisability regarding interventions to improve continence, and to explore health professionals' and school and social care staff's perspectives regarding factors affecting intervention use.

Confidential: For Review Only

Methods

Survey development

Survey questions and response options were developed in collaboration with the ICoN Family Faculty public involvement group, Professional Advisory Group, and young people with neurodisability. Surveys were developed through iteration and piloting.

The surveys included adaptive questioning; respondents were only asked to answer questions that were relevant to them. Parent carers, health professionals (HP) and school and social care staff (SSC) completed an initial eligibility question (related to their experience of continence support and neurodisability), and chose a clinical group about which to complete the survey; non-spinal cord pathology, defined as *social/communication or attention/behaviour difficulty, learning disability or physical or movement disability*, or spinal cord pathology, defined as *bladder and/or bowel impairment due to damage to the spinal cord e.g., neurogenic/neuropathic condition.* HPs had the option to complete the survey for both clinical groups.

Question order was fixed within surveys, following a logical order in terms of the respondent characteristics and experiences. Surveys included a maximum of five demographic characteristics questions, and up to 14 questions about professional practice and/or experience regarding interventions for improving continence. The children and young people's survey included just four questions regarding toileting abilities and experiences. The HP and SSC surveys included 16 additional questions regarding service provision, which all respondents, regardless of chosen clinical group, were asked to complete. HP survey questions reported in this paper come from the service provision section, not separated by clinical group. Box 1 lists the survey questions covered in this paper.

Participants and procedure

Parent carers, children and young people, HPs, and SSC were invited to participate via a study advert, shared by over 100 societies, charities, organisations, professional contacts, schools, local authorities and national parent carer and family forums. Study information was also advertised regularly by the research team, via social media. A copy of the study advert can be found in the supplementary material. This targeted recruitment gathered a diverse sample of participants with knowledge and experience of continence services and interventions for children and young people with neurodisability.

Participants registered via a study website (https://blogs.exeter.ac.uk/iconstudy/) to be emailed a personalised link to complete the relevant survey. This registration process created a 'closed'

Confidential: For Review Only

survey, allowing the study team to know to whom the invitations were sent, rather than an open survey for anyone to answer. Monitoring of registered email addresses allowed identification of duplicated registrations, and up to four reminders were sent to non-responders. Each participant was assigned a unique identifier via the registration website, preventing participants from completing the survey more than once. No analysis of IP addresses or log files was conducted.

Informed consent was assumed through voluntary registration online (11). Participants were encouraged to read the participant information sheet available via the registration webpage. Participation was voluntary, and no participation incentive was offered. The time required to complete the survey was not recorded although participants were advised that the survey required mostly tick box responses and would take approximately 15 minutes. Participants were able to review and save their responses prior to submission. All individually identifiable data were password protected via the registration website.

Data collection and reporting

The surveys were open between July and December 2019.

Numbers of registrations and completed surveys were recorded. Data were exported into Microsoft Excel and analysed using R (12). Guidelines for reporting online surveys were followed (13)), see supplementary material for a copy of the completed CHERRIES checklist. The number and percentage of completed surveys (as a proportion of personalised links sent) are reported. Demographic characteristics are summarised and the number of responses to each survey question reported. As not all questions were mandatory, the total number of responses varies across questions. Responses of 'don't know', 'never used', 'no knowledge' or 'never happened to me' regarding a service or intervention, are excluded from the description of results but are included in results tables. Parent carer survey responses are reported by clinical group. HP survey responses are reported by job role. Selected free-text responses illustrate salient results from the numerical data.

Ethics approval

The University of Exeter Medical School Research Ethics Committee approved the procedures (UEMS REC 19/B/199).

Confidential: For Review Only

Results

Survey responses

In total, 605 responses were received from 1028 parent carer registrations, 20 from 26 registered children and young people, 202 from 352 HP registrations, and 122 from 202 SSC registrations. Nine responses were excluded from both the parent carer and HP survey and three from the SSC survey due to ineligibility (no experience of continence support for, or the care of, children and young people with neurodisability). A further 17 parent carer responses were excluded because their child did not belong to one of the two clinical groups (n = 10) or could not be allocated to one of these groups from their free-text description of their child's condition (n = 7). Completed survey responses are therefore reported from 579 (56.3%) parent carers, 20 (77%) children and young people, 193 (54.8%) HPs, and 119 (58.9%) SSC.

Characteristics of respondents

Ninety percent (522/579) of parent carers were the child's mother, 396 (68.4%) of the children about whom the parent carers were completing the survey were male, and 446 (77%) were aged between 5 and 17 years. Over two thirds of children and young people respondents were female (14/20), of white ethnic origin (19/20), aged over 18 years (14/20) and were identified as being in the non-spinal cord pathology clinical group (17/20) from a free-text description of their condition. HPs were grouped into six categories by job role: bladder and bowel specialist (BBS) nurse; nurse; paediatrician; surgeon; therapist; other. Details of the roles included within these categories are provided elsewhere (9). Common SSC roles were teachers (29/119, 24.4%), assistants (28/119, 23.5%) and 'others' (36/119, 30.3%), and almost half worked in special schools (51/119, 42.9%). All geographical regions of England were represented (Table 1).

Difficulties in helping children and young people use interventions: parent carers' and school and social care staff's perspectives

The pattern of responses from parent carers regarding difficulties in helping children and young people to use toileting methods was similar for both clinical groups. The most common difficulties reported were the child's lack of understanding (337/556, 60.6%) and willingness (343/556, 61.7%). Regarding services, the most frequently reported difficulties were access to appropriate help (240/556, 43.2%); lack of consistency of support in different environments (209/556, 37.6%); delays in professional assessments (151/556, 27.2%); and lack of funding for equipment and products (129/556, 23.2%). The most common SSC-reported difficulties included children and young people's lack of understanding of what was required (67/115,

Confidential: For Review Only

58.3%), limited parent carer capability and time (70/115, 60.9%), and lack of appropriate facilities at school (69/115, 60%) (Table 2).

Acceptability of interventions: children's and young people's perspectives

Most children and young people with experience of toileting interventions felt happy or OK about using a hoist or frame (8/10), an alarm or timer (6/9), and half felt positively about using continence products and medications (6/12 for both). In contrast, many indicated unhappiness with following water/food diets (9/16), using a catheter or bowel washout (5/8) or having surgery (8/10). Most children and young people felt happy or OK using the toilet at home (14/18), but many felt negatively about using toilets at school, college or work (8/18), and out and about (14/19) (Table 3).

Ease of use of interventions: parent carers' and school and social care staff's perspectives

Parent carers reported that toileting products (320/401, 80%) and simple aids/equipment (216/292, 74%) were easiest to use in the non-spinal cord pathology group. In the spinal cord pathology group, all five parent carers with experience of housing adaptations, and 15/16 with experience of continence products, reported them to be easy or very easy to use.

A large proportion of SSC with experience of interventions to promote continence found toileting products or training methods easy or very easy to use; dietary advice (76/96, 79%), fluid intake advice (88/106, 83%), and behavioural interventions (98/109, 89.9%). In contrast, only 31% of parent carers (93/299) found behavioural interventions easy or very easy to use. (Supplementary Material Table 1).

Availability of interventions: parent carers', school and social care staff's and health professionals' perspectives

Substantial numbers of HPs with knowledge of specific aids, said they were not available or only available by purchase (behavioural aids such as alarms, 74/144 (51.4%); simple aids such as raised seats or steps, 71/146 (48.6%); and housing adaptations, 39/124 (31.4%)). The most common response regarding provision of continence products was that they were supplied free of charge (17/18, 94% bladder and bowel specialist (BBS) nurses; 11/17, 65% therapists; 55/93, 59% SSC; 256/374, 68.4% and 9/17, 53% parent carers for non-spinal cord and spinal cord pathologies, respectively) (Supplementary Material Table 2).

Acceptability of waiting times for continence support: parent carers' and health professionals' perspectives

Confidential: For Review Only

Roughly half of parent carers and HPs with knowledge of waiting times for continence support considered them acceptable (Table 4). Parent carers and HPs who considered waiting times to be unacceptable, described long waits for assessments, lack of funding and staffing:

> *"Waiting times are too long for assessment, and then provision of complex equipment and/or adaptations can also take a long time". [Therapist 48880209]*
>
> *"Not enough continence support so waiting times long and services are not funded". [Nurse 49177881]*
>
> *"Everything takes a very long time to come through, including assessment - not had one for years". [Parent carer 49575343]*
>
> *"Two part time staff for the whole county, very few appointments and long waiting list. Very restrictive criteria to access the service". [Parent carer 50843591]*
>
> *"Funding is always an issue. It's always a fight to get appropriate services" [Parent carer 50044306]*

Facilitators and barriers to improving continence: health professionals' perspectives

HPs from all roles reported that funding frequently adversely affected provision of continence services. Many reported the lack of a dedicated continence service or a failure to provide a specific service in their area that was known to be available elsewhere. Ninety-one percent (142/156) of HPs reported lack of funding and/or resources as barriers to provision of dedicated continence services (Table 5).

HP perspectives varied on whether provision of pads/nappies was a barrier or enabler to toilet training. Excluding those who didn't know, 16/20 BBS nurses, approximately 2 in 3 nurses, just over half of therapists, half of 'other' professionals, 7/17 paediatricians and 1/7 surgeons, considered it a barrier (Table 6). Reasons included:

> *"The continence pads don't allow the child to feel wet therefore this reduces their understanding of doing wees and poos". [Nurse 49013158]*
>
> *"For some children it is a barrier as they become dependent on the security of the product and are less motivated to try toileting programmes. This is also age dependent - more successful when the toileting programmes are started earlier (chronological age rather than developmental age)". [Nurse 49176801]*

Confidential: For Review Only

Discussion

This study explored the use, availability and acceptability of continence interventions for children and young people with neurodisability. Difficulties with implementing interventions identified by parent carers were consistent across clinical groups, reporting that the child was not ready, unable to learn or had inadequate understanding. SSC reported capability and time of the parent carer and lack of consistency of support across environments as key difficulties. Similarly, an earlier study has highlighted the importance of parent training as a key aspect of toilet training (14). The lack of adequate and accessible public facilities highlighted by respondents as a barrier to toileting, emphasises the need for more accessible and/or specialist toilets; 'Changing Places' toilets are an example which provide necessary equipment and appropriate space in a clean and safe environment to support people with physical disabilities when outside home (14).

Children and young people were generally unhappy about using many toileting interventions, particularly diets or catheter/bowel washouts. They also reported that using the toilet at home was preferable to toilets in other environments, perhaps due to inadequacy of other facilities as highlighted by SSC, and the important role the home plays in providing privacy for children and young people with disabilities (15). A review by Brazzelli et al (2011) also emphasised how medical or behavioural interventions can be upsetting for children and young people and therefore acceptability should be a key consideration in provision. Early introduction of these interventions has been shown to significantly improve acceptability to both children and young people and parents (16), and as many children and young people with neurodisability may not reach a level of understanding or willingness or reach a level of developmental ability to allow them to achieve continence, early education of parents and introduction of continence strategies is important. Our survey results only touched on acceptability of interventions, and this is an area that warrants further research (17).

In contrast to the lack of acceptability of interventions indicated by children and young people, SSC said that diet and fluid intake advice, behavioural interventions, physical aids, and continence products were easy to implement. Parent carers and SSC also reported continence products as easy to use, and HPs reported that they were typically provided free of charge, as recommended in NHS guidelines on managing incontinence in children with neurological disability (18). Some HPs, SSC and parent carers, however, did indicate that products were not

https://mc.manuscriptcentral.com/bmjpo

Confidential: For Review Only

free in their area, with many HPs also suggesting that provision of products was a barrier to achieving continence. Containment products do not necessarily improve continence in the same way as medical or behavioural-based toilet training interventions do, which aim for the child to become 'clean and dry'. Impairment of children and young people with spinal cord pathology however, may mean achieving continence is not a realistic goal and therefore the use of continence products is necessary, which can be at a considerable cost if not provided through health services (10,18). Recent research highlighted that provision of continence pads is inadequate and *ad hoc* (19,20), perhaps due to the influence that commissioning and funding arrangements have on provision, as highlighted in the HP survey. Research has also shown that children in low income families are four times more likely to be urinary incontinent than children in high income families (21) so those families that cannot afford continence products more likely experience compromised comfort and dignity.

Waiting times and funding were highlighted as influencing implementation of interventions. HPs had divided opinions on acceptability of waiting times to receive support and reported a lack of funding and dedicated services impacting provision. Similarly, parent carers reported variable waiting times, and described difficulties accessing appropriate support. This is a key finding as, coupled with a lack of continence product provision, it likely impacts the quality of life of the child and their family; similar findings were highlighted in studies of paediatric referrals for toileting support (22,23). Research by Kroeger and Sorensen-Burnworth (24) emphasised that incontinence is a significant limiter of quality of life for those with developmental disabilities and difficulties in accessing adequate support in a timely manner is likely to significantly amplify the impact. Early intervention programmes can enable children to learn and develop age-appropriate toileting skills, however, age-related criteria in many areas for referral, means that opportunities for early intervention may be missed.

Strengths of an online survey methodology included the potential to reach a large, geographically and demographically diverse sample, at low cost and ease for participants, as demonstrated in other studies (25,26). Our surveys were developed and piloted with a diverse range of relevant stakeholders, parent carers and young people with neurodisability, ensuring that questions were relevant. Limitations included: the registration process led to potential delays in responses and risk of participants entering contact details incorrectly; partial response, perhaps due to the length of the survey; and responses were limited to those willing and able to use an online method. While there was an option for children and young people to have help

Confidential: For Review Only

to complete their survey, there was still a requirement for them to be able to express their feelings/experiences, meaning that only those with sufficient developmental ability to self-report were captured. Parent carers were not asked in the survey to report if their child had sufficient developmental capacity to adhere to an intervention. Therefore, where parent carers have reported their child's lack of willingness as a difficulty in helping them to use toileting methods, it is not possible to distinguish whether the child they are reporting about has sufficient developmental capacity to be willing or not. Other limitations included that although data presented are from across England, the South West is over-represented, with this region having the largest proportion of respondents out of all of the regions, for all surveys. The survey findings may not adequately reflect variation in service provision and experience by locality, so can therefore only provide a snapshot of current practice. Overall, 93% of parent carers and children and young people who completed the surveys identified themselves as White, which is higher than the current census data in which 81.7% of usual residents in England and Wales identified as being in the White ethnic category (27). Finally, surveys were completed prior to the COVID-19 pandemic, so findings may be different when restrictions placed on health services during the pandemic are considered.

The survey findings highlighted perceived ability of the child and lack of adequate facilities as key factors affecting implementation of toileting interventions for children and young people with neurodisability. This emphasises the requirement of proactive and timely individual assessments of need, to facilitate the introduction of an appropriate individualised toileting skills development programme, to give the children and young people who have the appropriate physical and developmental ability, the opportunity to become toilet trained or at least clean and dry at the same age as their typically developing peers. Previous guidance and research have also highlighted this need (24,28).

The impact of incontinence on quality of life has previously been highlighted (24,29) and our findings reinforce the need for further research around availability and acceptability of toileting interventions to adequately support children and young people with neurodisability. Effectiveness of interventions is likely to impact opinions of acceptability; whilst the surveys gathered some information on perceived effectiveness (10), further evidence would add to the knowledge around appropriate incontinence support for children and young people with neurodisability.

Confidential: For Review Only

Funding statement

This project was funded by the National Institute for Health and Care Research (NIHR) Health Technology Assessment programme (Award ID: 17/20/02). Helen Eke, Susan Ball, Stuart Logan, Rob Anderson, Harriet Hunt, Jo Thompson Coon, Rebecca Whear and Christopher Morris have previously been or are currently funded or supported by the NIHR Applied Research Collaboration South West Peninsula. The views expressed are those of the authors and not necessarily those of the NIHR or the Department of Health and Social Care.

Competing interests

The authors Helen Eke, Susan Ball, Annette Allinson, Rob Anderson, Harriet Hunt, Eve Hutton, Claire Lindsay, Stuart Logan, Julia Melluish, June Rogers, Jo Thompson Coon, Rebecca Whear, and Christopher Morris declare no competing interests.

Anne Wright is an Educational Chairperson of the International Children's Continence Society (not reimbursed), President of the charity ERIC (not reimbursed), and participates in the advisory board for Astellas DSMB Mirabegron.

Davina Richardson is employed by the charity Disabled Living, which receives unrestricted funding grants from the various commercial organisations to support the work of the charity. She is paediatric advisor to the Executive of the Association for Continence Advice, and is co-vice chair of the Paediatric Continence Forum (PCF), which is an independent multidisciplinary group of healthcare professionals campaigning for better care of bladder and bowel problems in children and young people, with a particular focus on community care.

Nick Madden is Honorary Secretary for Sutton and District Medical Society, and was previously vice-chair for the PCF.

Author contributions

CM was the chief investigator and is guarantor. SB, AA, RA, CL, SL, NM, JM, DR, JR, JTC and AW were coinvestigators, and HE, HH and RW were research fellows on the grant application to the NIHR. All authors were-involved in the development of the surveys. HE led the development of the surveys and the data collection. SB was statistical lead and conducted the analyses. HE and SB led the writing of the manuscript. All authors reviewed and revised the manuscript and approved the final manuscript.

Data sharing

Confidential: For Review Only

Data are available upon reasonable request. The full report of all survey findings is available from Open Research Exeter (ORE), https://ore.exeter.ac.uk/repository/handle/10871/127283.

Confidential: For Review Only

References [29/30]

1. Morris C, Janssens A, Tomlinson R, Williams J, Logan S. Towards a definition of neurodisability: A Delphi survey. Dev Med Child Neurol. 2013 Dec;55(12):1103–8.
2. Järvelin MR. Developmental history and neurological findings in enuretic children. Dev Med Child Neurol. 2008 Nov 12;31(6):728–36.
3. Foreman DM, Thambirajah MS. Conduct disorder, enuresis and specific developmental delays in two types of encopresis: a case-note study of 63 boys. Eur Child Adolesc Psychiatry. 1996 Apr;5(1):33–7.
4. Wendt L, Similä S, Niskanen P, Järvelin MR. Development of bowel and bladder control in the mentally retarded. Dev Med Child Neurol. 2010 Aug 24;32(6):515–8.
5. von Gontard A, de Jong TPVM, Rantell A, Nieuwhof-Leppink A, Badawi JK, Cardozo L. Do we manage incontinence in children and adults with special needs adequately? ICI-RS 2014. Neurology and Urodynamics. 2016 Feb;35(2):304–6.
6. Townsend J, Heng L, Thomas T, Egan M, Meade T. Costs of incontinence to families with severely handicapped children. J Public Health (Bangkok). 1981;3(2):119–22.
7. Von Gontard A, Nevéus T. The management of disorders of bladder and bowel control in childhood. Mac Keith Press; 2006.
8. Klassen TP, Kiddoo D, Lang ME, Friesen C, Russell K, Spooner C, et al. The effectiveness of different methods of toilet training for bowel and bladder control. Evidence Report / Technology Assessment (Full Report). 2006 Dec;(147):1–57.
9. Morris C, Simkiss D, Busk M, Morris M, Allard A, Denness J, et al. Setting research priorities to improve the health of children and young people with neurodisability: a British Academy of Childhood Disability-James Lind Alliance Research Priority Setting Partnership. BMJ Open. 2015;5.
10. Eke H, Hunt H, Ball S, Rogers M, Whear R, Allinson A, et al. Improving continence in children and young people with neurodisability: a systematic review and survey. Health Technol Assess (Rockv). 2021 Nov;25(73):1–258.
11. Central University Research Ethics Committee (CUREC). Internet-Mediated Research [Internet]. 2021 Feb. Available from: http://ukrio.org/wp-content/uploads/UKRIO-Guidance-Note-Internet-Mediated-Research-v1.0.pdf
12. R Core Team. R: A Language and Environment for Statistical Computing [Internet]. Vienna, Austria: R Foundation for Statistical Computing; 2019. Available from: https://www.R-project.org/
13. Eysenbach G. Improving the quality of Web surveys: the Checklist for Reporting Results of Internet E-Surveys (CHERRIES). J Med Internet Res. 2004 Sep 29;6(3):e34.
14. Changing Places Consortium. What are Changing Places toilets? [Internet]. 2022 [cited 2022 Jun 14]. Available from: https://www.changing-places.org/
15. Weigel-Garrey C, Cook C, Brotherson M. Children and Privacy. Choice, Control, and Access in Home Environments. J Fam Issues. 1998;19(1):43–64.
16. Bauer SB, Saunders RA, Masoom SN, Choung K, Hayes LC, Price DE, et al. The art of introducing clean intermittent catheterization: How families respond and adapt: A qualitative study. Neurourol Urodyn. 2023 Jan 9;42(1):309–21.
17. Brazzelli M, Griffiths P V, Cody JD, Tappin D. Behavioural and cognitive interventions with or without other treatments for the management of faecal incontinence in children. Cochrane Database Syst Rev. 2011 Dec 7;(12).

Confidential: For Review Only

18. National Institute of Health and Care Excellence. Urinary incontinence in neurological disease: assessment and management Clinical guideline [Internet]. 2012. Available from: https://www.nice.org.uk/guidance/cg148
19. Murphy C, et al. Continence pad provision: meeting patients' fundamental care needs. Nursing Times (online) [Internet]. 2019 [cited 2022 Jun 14];115(2):39–42. Available from:https://www.nursingtimes.net/clinical-archive/continence/continence-pad-provision-meeting-patients-fundamental-care-needs-28-01-2019/
20. Richardson D. Guidance for the provision of continence containment products to children and young people. A consensus document. [Internet]. 2021 [cited 2022 Jun 14]. Available from: https://www.bbuk.org.uk/wp-content/uploads/2021/11/Guidance-for-the-provision-of-continence-containment-products-to-children-2021-2.pdf
21. Savaser S, Kizilkaya Beji N, Aslan E, Gozen D. The Prevalence of Diurnal Urinary Incontinence and Enuresis and Quality of Life: Sample of School. Pediatric Urology [Internet]. 2018 [cited 2023 Oct 16];15(4):173–9. Available from: https://journals.sbmu.ac.ir/urolj/index.php/uj/article/view/3982/1448
22. Trajanovska M, Liew A, Gibb S, Goldfeld S, King SK. Retrospective audit of referral and triage pathways of paediatric patients with constipation and soiling. J Paediatr Child Health. 2020 Feb 1;56(2):298–303.
23. Eiselen C, Trajanovska M, Griffith A, Phan T, Goldfeld S, Gibb S, et al. Audit of enuresis referrals on the waiting list for a tertiary hospital outpatient clinic. J Paediatr Child Health. 2021 Oct 1;57(10):1645–50.
24. Kroeger KA, Sorensen-Burnworth R. Toilet training individuals with autism and other developmental disabilities: A critical review. Vol. 3, Research in Autism Spectrum Disorders. 2009. p. 607–18.
25. Lyons AC, Cude B, Lawrence FC, Gutter M. Conducting Research Online: Challenges Facing Researchers in Family and Consumer Sciences. Fam Consum Sci Res J. 2005;33(4):341–56.
26. Solomon DJ. Conducting web-based surveys. Practical Assessment, Research, and Evaluation. 2000;7(19).
27. Office for National Statistics (ONS). Ethnic group, England and Wales: Census 2021 [Internet]. 2022 Nov [cited 2023 Oct 16]. Available from: https://www.ons.gov.uk/peoplepopulationandcommunity/culturalidentity/ethnicity/bulletins/ethnicgroupenglandandwales/census2021#cite-this-statistical-bulletin
28. Kroeger K, Sorensen R. A parent training model for toilet training children with autism. Journal of Intellectual Disability Research. 2010 Jun;54(6):556–67.
29. Ranasinghe N, Devanarayana NM, Benninga MA, Van Dijk M, Rajindrajith S. Psychological maladjustment and quality of life in adolescents with constipation. Arch Dis Child. 2017 Mar 1;102(3):268–73.

Confidential: For Review Only

Survey questions included in this paper

Parent carers

- What difficulties have you found using methods to help with toileting at home?
- If you and your child have experienced any of the following methods to help with toileting, please indicate how easy you found it using them at home.
- Where you live, please indicate which toileting products or equipment are provided for families of children and young people with special educational needs and/or a disability.
- Where you live, how acceptable is the waiting time for families to get the equipment and products they require once an assessment has been completed?

Children and young people

- How do you feel about . . . (list of different interventions)
- How do you feel about using the toilet … (list of different environments)

Health professionals

- Where you work, please indicate which toileting products or equipment are provided for families of children and young people with special educational needs and/or a disability.
- In your opinion, how acceptable is the waiting time in your area for families to receive the toileting support or equipment/products they require?
- In your opinion, to what extent do local commissioning/funding arrangements influence the toileting support you offer for individual children and young people with special educational needs and/or a disability (e.g., would you recommend the use of continence pads as they are supplied free of charge for families locally)?
- Where you work, do you have a bladder and bowel protocol/pathway for children and young people with special educational needs and/or a disability?
- Where you work, are there any toileting interventions for children and young people with special educational needs and/or a disability that are not provided, which you know are provided elsewhere?
- In your opinion, what do you think is the main barrier to developing a dedicated bladder and bowel pathway or service?
- In your opinion, is the provision of continence pads for children and young people with special educational needs and/or a disability a barrier or enabler for achieving continence?

School and social care staff

- Where you work, what difficulties have you found in helping children and young people to use the toileting methods (e.g., alarms or frames) provided?
- Where you work, how easy is it for you to provide or use the following methods to help children and young people with toileting?
- Where you work, please indicate which toileting products or equipment are provided for families of children and young people with special educational needs and/or a disability.

Box 1: List of survey questions included in analyses

Confidential: For Review Only

Table 1: Characteristics of survey respondents. Values are n (%) in respondent group

Parent carers		Children and young people		Health professionals		School and social care staff	
**Characteristic**	**N = 579**	**Characteristic**	**N = 20**	**Characteristic**	**N = 193**	**Characteristic**	**N = 119**
Child's gender		***Gender***		***Gender***		***Gender***	
Male	396 (68.4)	Male	6 (30)	Male	16 (8.3)	Male	6 (5.0)
Female	181 (31.3)	Female	14 (70)	Female	158 (81.9)	Female	111 (93.3)
pnts	2 (0.3)	pnts	0 (0)	pnts	7 (3.6)	pnts	1 (0.8)
na	0 (0)	na	0 (0)	na	12 (6.2)	na	1 (0.8)
Region of England where live		***Region of England where live***		***Region of England where work***		***Region of England where work***	
North West	40 (6.9)	North West	3 (15)	North West	21 (10.9)	North West	10 (8.4)
North East	21 (3.6)	North East	1 (5)	North East	12 (6.2)	North East	4 (3.4)
Y & H	31 (5.4)	Y & H	2 (10)	Y & H	9 (4.7)	Y & H	5 (4.2)
East Midlands	32 (5.5)	East Midlands	0 (0)	East Midlands	9 (4.7)	East Midlands	2 (1.7)
West Midlands	53 (9.2)	West Midlands	0 (0)	West Midlands	7 (3.6)	West Midlands	3 (2.5)
East Anglia	26 (4.5)	East Anglia	1 (5)	East Anglia	9 (4.7)	East Anglia	2 (1.7)
South West	166 (28.7)	South West	4 (20)	South West	39 (20.2)	South West	60 (50.4)
South East	123 (21.2)	South East	4 (20)	South East	26 (13.5)	South East	22 (18.5)
London	48 (8.3)	London	1 (5)	London	38 (19.7)	London	6 (5.0)
Outside England	28 (4.8)	Outside England	4 (20)	Outside England	18 (9.3)	Outside England	2 (1.7)
na	11 (1.9)	na	0 (0)	na	5 (2.6)	na	3 (2.5)
Age of child (years)		***Age (years)***				***Job role***	
Under 5	68 (11.7)	Under 5	0 (0)			Manager	19 (16.0)
5-7	136 (23.5)	5-7	0 (0)			Assistant	28 (23.5)
8-11	153 (26.4)	8-11	1 (5)			Teacher	29 (24.4)
12-17	157 (27.1)	12-17	5 (25)			Enabler	5 (4.2)
18-25	64 (11.1)	18-25	11 (55)			Social worker	0 (0)
Over 25	0 (0.0)	Over 25	3 (15)			Therapist	1 (0.8)
na	1 (0.2)	na	0 (0)			Other	36 (30.3)
Ethnicity		***Ethnicity***				na	1 (0.8)
White	540 (93.3)	White	19 (95)			***Where work***	
Mixed	13 (2.2)	Mixed	0 (0)			Mainstream school	30 (25.2)
Asian	10 (1.7)	Asian	0 (0)			Special school	51 (42.9)
Black	8 (1.4)	Black	0 (0)			Hospice / Respite care	0 (0)
Other	6 (1.0)	Other	1 (5)			Short break facility	5 (4.2)
na	2 (0.3)	na	0 (0)			Community care	6 (5.0)
Relationship to child		***How completed survey***				Primary care	1 (0.8)

Confidential: For Review Only

Mother*	522 (90.2)	On my own	17 (85)	Secondary care	0 (0)
Father	25 (4.3)	Someone is helping me	2 (10)	Other	25 (21.0)
Parent**	15 (2.6)	Someone is doing it for me	1 (5)	na	1 (0.8)
Grandparent	10 (1.7)	na	0 (0)		
Carer	2 (0.3)				
na	5 (0.9)				

pnts = prefer not to say; na = not answered; Y & H = Yorkshire & Humberside; * = including foster, adoptive and step mother; ** = including foster parent

Confidential: For Review Only

Table 2: Parent carers' and school and social care staff's perspectives on what difficulties they have found in helping children and young people to use toileting methods. Note: respondents were asked to choose all response options that applied

Response option, n (% of respondents)	Parent carers				School and social care staff	
	Non-spinal cord pathology		Spinal cord pathology			
	N = 536		N = 20		N = 115	
No difficulties experienced	34	(6)	4	(20)	16	(14)
Child/young person's knowledge and understanding of what is required	328	(61)	9	(45)	67	(58)
Parent carer knowledge and understanding of what is required	56	(10)	4	(20)	54	(47)
Parent carer ability and time to focus on toileting	164	(31)	6	(30)	70	(61)
Child/young person's willingness*	329	(61)	14	(70)	-	-
Child/young person's adherence to the intervention**	-	-	-	-	48	(42)
Parent carer and child's lack of interest / motivation to change	67	(13)	1	(5)	40	(35)
Not enough training in how to use the methods offered*	52	(10)	1	(5)	-	-
Delays in professional assessments	149	(28)	2	(10)	44	(38)
Access to appropriate help and support	233	(43)	7	(35)	38	(33)
Funding and/or resources for equipment and products	126	(24)	3	(15)	53	(46)
Lack of consistency in support in different environments e.g., facility at home but not at school	205	(38)	4	(20)	69	(60)
Other	47	(9)	1	(5)	5	(4)

* Response option included in parent carer survey only.

** Response option included in school and social care staff survey only.

Confidential: For Review Only

Table 3: Children and young people's perspectives on how they feel about toileting interventions and about using the toilet in different places

**n (% of respondents)**	**Very happy**		**OK**		**A bit unhappy**		**Very unhappy**		**Don't know OR this has never happened to me**	
Intervention										
Following a special water/food diet (N = 20)	2	(10)	5	(25)	7	(35)	2	(10)	4	(20)
Using an alarm or timer to remind me to wee/poo (N = 20)	0	(0)	6	(30)	1	(5)	2	(10)	11	(55)
Using a hoist or a frame to help me use the toilet (N = 20)	1	(5)	7	(35)	1	(5)	1	(5)	10	(50)
Using pads, nappies or pull ups (N = 20)	3	(15)	3	(15)	1	(5)	5	(25)	8	(40)
Taking medication to help me wee/poo (N = 20)	4	(20)	2	(10)	5	(25)	1	(5)	8	(40)
Using a catheter or bowel washout (tubes that help you to wee or poo) (N = 20)	1	(5)	2	(10)	0	(0)	5	(25)	12	(60)
Having surgery (N = 20)	2	(10)	0	(0)	2	(10)	6	(30)	10	(50)
Place										
Using the toilet at home (N = 19)	12	(63)	2	(11)	3	(16)	1	(5)	1	(5)
Using the toilet at school / college / work (N = 20)	5	(25)	5	(25)	3	(15)	5	(25)	2	(10)
Using the toilet when out and about e.g., in a restaurant (N = 20)	2	(10)	3	(15)	9	(45)	5	(25)	1	(5)

Numbers are n (%) of respondents who gave an answer to the statement, so percentages add up to 100 across the rows.

Confidential: For Review Only

Table 4: Parent carers' and health professionals' perspectives on the acceptability of waiting times for families to receive the required toileting support or equipment/products

	**Parent carers**				**Health professionals**											
**Response option, n (% of respondent group)**	**Non-spinal cord pathology**		**Spinal cord pathology**		**BBS Nurses**		**Nurses**		**Paediatricians**		**Surgeons**		**Therapists**		**Other**	
	**N = 553**		**N = 20**		**N = 22**		**N = 79**		**N = 30**		**N = 15**		**N = 33**		**N = 13**	
Very acceptable	27	(5)	3	(15)	4	(18)	5	(6)	0	(0)	2	(13)	2	(6)	0	(0)
Acceptable	121	(22)	7	(35)	8	(36)	32	(41)	11	(37)	3	(20)	9	(27)	7	(54)
Unacceptable	79	(14)	3	(15)	7	(32)	17	(22)	9	(30)	7	(47)	11	(33)	4	(31)
Very unacceptable	64	(12)	2	(10)	2	(9)	9	(11)	2	(7)	1	(7)	1	(3)	1	(8)
Don't know	262	(47)	5	(25)	1	(5)	16	(20)	8	(27)	2	(13)	10	(30)	1	(8)

BBS = Bladder and Bowel Specialist.

Confidential: For Review Only

Confidential: For Review Only

Table 5: Health professionals’ perspectives on: (a) the extent to which local commissioning/funding arrangements influence the toileting support they offer for children and young people with neurodisability; (b) whether, where they work, there is a bladder and bowel protocol/pathway for children and young people with neurodisability; (c) whether there are any toileting interventions for children and young people with neurodisability that are not provided, which they know are provided elsewhere; (d) what they think is the main barrier to developing a dedicated bladder and bowel pathway or service. Responses to (d) are shown for those health professionals who answered either ‘yes’ or ‘no’ to the question of whether there is a bladder and bowel protocol/pathway for children and young people with neurodisability - those who answered ‘don’t know’ are excluded

**Question**	**Response option, n (% of respondents in role)**	**BBS Nurses**		**Nurses**		**Paediatricians**		**Surgeons**		**Therapists**		**Other**	
		**N = 22**		**N = 79**		**N = 31**		**N = 15**		**N = 33**		**N = 13**	
(a) To what extent do local commissioning/funding arrangements influence the toileting support that you offer?	Never	5	(23)	12	(15)	2	(6)	2	(13)	6	(18)	1	(8)
	Sometimes	6	(27)	18	(23)	10	(32)	5	(33)	8	(24)	5	(38)
	Often	3	(14)	11	(14)	10	(32)	4	(27)	5	(15)	1	(8)
	Always	6	(27)	19	(24)	6	(19)	1	(7)	1	(3)	1	(8)
	Don't know	2	(9)	19	(24)	3	(10)	3	(20)	13	(39)	5	(38)
		**N = 22**		**N = 79**		**N = 30**		**N = 15**		**N = 33**		**N = 13**	
(b) Is there a bladder and bowel protocol/pathway?	Yes	17	(77)	46	(58)	10	(33)	10	(67)	5	(15)	5	(38)
	No	4	(18)	21	(27)	17	(57)	4	(27)	13	(39)	4	(31)
	Don’t know	1	(5)	12	(15)	3	(10)	1	(7)	15	(45)	4	(31)

(c) are there any toileting interventions that are not provided, which they know are provided elsewhere?		**N = 22**		**N = 79**		**N = 30**		**N = 15**		**N = 33**		**N = 13**	
	Yes	6	(27)	18	(23)	8	(27)	6	(27)	18	(23)	8	(27)
	No	11	(50)	26	(33)	4	(13)	11	(50)	26	(33)	4	(13)
	Don’t know	5	(23)	35	(44)	18	(60)	5	(23)	35	(44)	18	(60)
(d) what is the main barrier to developing a dedicated bladder and bowel pathway or service		**N = 21**		**N = 67**		**N = 27**		**N = 14**		**N = 18**		**N = 9**	
	Lack of funding and/or resources	16	(76)	38	(57)	17	(63)	16	(76)	38	(57)	17	(63)
	Lack of professional interest	1	(5)	9	(13)	2	(7)	1	(5)	9	(13)	2	(7)
	Time	0	(0)	10	(15)	3	(11)	0	(0)	10	(15)	3	(11)
	Lack of need in local area	0	(0)	1	(1)	2	(7)	0	(0)	1	(1)	2	(7)
	Don’t know	1	(5)	6	(9)	1	(4)	1	(5)	6	(9)	1	(4)
	Other	3	(14)	3	(4)	2	(7)	3	(14)	3	(4)	2	(7)

BBS = Bladder and Bowel Specialist.

Confidential: For Review Only

Table 6: Health professionals' perspectives on whether provision of continence pads for children and young people with special educational needs and/or a disability is a barrier or enabler for achieving continence

Response option, n (% of respondents in role)	BBS Nurses		Nurses		Paediatricians		Surgeons		Therapists		Other	
	N = 22		N = 76		N = 30		N = 15		N = 33		N = 13	
Barrier	16	(73)	39	(51)	7	(23)	1	(7)	9	(27)	5	(38)
Enabler	4	(18)	18	(24)	10	(33)	6	(40)	7	(21)	5	(38)
Don't know	2	(9)	19	(25)	13	(43)	8	(53)	17	(52)	3	(23)

BBS = Bladder and Bowel Specialist.

Confidential: For Review Only

Recruiting now!

We are conducting an online survey about toileting with health professionals, parent/carers, young people and education and social care staff.

The NHS wants to improve toileting services for children and young people with special educational needs and disability aged up to 25 years. We need to gather the views and experiences of families, clinicians, and school and social care staff, to establish how toileting is managed currently. The results of the survey will help inform health and care services for children in the future.

For further information and to take part in the survey, visit:
http://sites.exeter.ac.uk/iconstudy/taking-part/

ICoN
improving continence for children and young people with neurodisability

FUNDED BY NIHR | National Institute for Health Research

For Review Only

Checklist for Reporting Results of Internet E-Surveys (CHERRIES)

Checklist Item	***Explanation***	***Page Number***
Describe survey design	Describe target population, sample frame. Is the sample a convenience sample? (In "open" surveys this is most likely.)	6
IRB approval	Mention whether the study has been approved by an IRB.	7
Informed consent	Describe the informed consent process. Where were the participants told the length of time of the survey, which data were stored and where and for how long, who the investigator was, and the purpose of the study?	7
Data protection	If any personal information was collected or stored, describe what mechanisms were used to protect unauthorized access.	7
Development and testing	State how the survey was developed, including whether the usability and technical functionality of the electronic questionnaire had been tested before fielding the questionnaire.	6
Open survey versus closed survey	An "open survey" is a survey open for each visitor of a site, while a closed survey is only open to a sample which the investigator knows (password-protected survey).	6
Contact mode	Indicate whether or not the initial contact with the potential participants was made on the Internet. (Investigators may also send out questionnaires by mail and allow for Web-based data entry.)	6
Advertising the survey	How/where was the survey announced or advertised? Some examples are offline media (newspapers), or online (mailing lists – If yes, which ones?) or banner ads (Where were these banner ads posted and what did they look like?). It is important to know the wording of the announcement as it will heavily influence who chooses to participate. Ideally the survey announcement should be published as an appendix.	6
Web/E-mail	State the type of e-survey (eg, one posted on a Web site, or one sent out through e-mail). If it is an e-mail survey, were the responses entered manually into a database, or was there an automatic method for capturing responses?	6
Context	Describe the Web site (for mailing list/newsgroup) in which the survey was posted. What is the Web site about, who is visiting it, what are visitors normally looking for? Discuss to what degree the content of the Web site could pre-select the sample or influence the results. For example, a survey about vaccination on a anti-immunization Web site will have different results from a Web survey conducted on a government Web site	6
Mandatory/voluntary	Was it a mandatory survey to be filled in by every visitor who wanted to enter the Web site, or was it a voluntary survey?	6
Incentives	Were any incentives offered (eg, monetary, prizes, or non-monetary incentives such as an offer to provide the survey results)?	6

Confidential: For Review Only

Time/Date	In what timeframe were the data collected?	7
Randomization of items or questionnaires	To prevent biases items can be randomized or alternated.	6
Adaptive questioning	Use adaptive questioning (certain items, or only conditionally displayed based on responses to other items) to reduce number and complexity of the questions.	6
Number of Items	What was the number of questionnaire items per page? The number of items is an important factor for the completion rate.	6
Number of screens (pages)	Over how many pages was the questionnaire distributed? The number of items is an important factor for the completion rate.	6
Completeness check	It is technically possible to do consistency or completeness checks before the questionnaire is submitted. Was this done, and if "yes", how (usually JAVAScript)? An alternative is to check for completeness after the questionnaire has been submitted (and highlight mandatory items). If this has been done, it should be reported. All items should provide a non-response option such as "not applicable" or "rather not say", and selection of one response option should be enforced.	7
Review step	State whether respondents were able to review and change their answers (eg, through a Back button or a Review step which displays a summary of the responses and asks the respondents if they are correct).	7
Unique site visitor	If you provide view rates or participation rates, you need to define how you determined a unique visitor. There are different techniques available, based on IP addresses or cookies or both.	N/A
View rate (Ratio of unique survey visitors/unique site visitors)	Requires counting unique visitors to the first page of the survey, divided by the number of unique site visitors (not page views!). It is not unusual to have view rates of less than 0.1 % if the survey is voluntary.	N/A
Participation rate (Ratio of unique visitors who agreed to participate/unique first survey page visitors)	Count the unique number of people who filled in the first survey page (or agreed to participate, for example by checking a checkbox), divided by visitors who visit the first page of the survey (or the informed consents page, if present). This can also be called "recruitment" rate.	8
Completion rate (Ratio of users who finished the survey/users who	The number of people submitting the last questionnaire page, divided by the number of people who agreed to participate (or submitted the first survey page). This is only relevant if there is a separate "informed consent" page or if the survey goes over several pages. This is a measure for attrition. Note that	8

Confidential: For Review Only

agreed to participate)	"completion" can involve leaving questionnaire items blank. This is not a measure for how completely questionnaires were filled in. (If you need a measure for this, use the word "completeness rate".)	
Cookies used	Indicate whether cookies were used to assign a unique user identifier to each client computer. If so, mention the page on which the cookie was set and read, and how long the cookie was valid. Were duplicate entries avoided by preventing users access to the survey twice; or were duplicate database entries having the same user ID eliminated before analysis? In the latter case, which entries were kept for analysis (eg, the first entry or the most recent)?	7
IP check	Indicate whether the IP address of the client computer was used to identify potential duplicate entries from the same user. If so, mention the period of time for which no two entries from the same IP address were allowed (eg, 24 hours). Were duplicate entries avoided by preventing users with the same IP address access to the survey twice; or were duplicate database entries having the same IP address within a given period of time eliminated before analysis? If the latter, which entries were kept for analysis (eg, the first entry or the most recent)?	7
Log file analysis	Indicate whether other techniques to analyze the log file for identification of multiple entries were used. If so, please describe.	7
Registration	In "closed" (non-open) surveys, users need to login first and it is easier to prevent duplicate entries from the same user. Describe how this was done. For example, was the survey never displayed a second time once the user had filled it in, or was the username stored together with the survey results and later eliminated? If the latter, which entries were kept for analysis (eg, the first entry or the most recent)?	6
Handling of incomplete questionnaires	Were only completed questionnaires analyzed? Were questionnaires which terminated early (where, for example, users did not go through all questionnaire pages) also analyzed?	7
Questionnaires submitted with an atypical timestamp	Some investigators may measure the time people needed to fill in a questionnaire and exclude questionnaires that were submitted too soon. Specify the timeframe that was used as a cut-off point, and describe how this point was determined.	7
Statistical correction	Indicate whether any methods such as weighting of items or propensity scores have been used to adjust for the non-representative sample; if so, please describe the methods.	7

This checklist has been modified from Eysenbach G. Improving the quality of Web surveys: the Checklist for Reporting Results of Internet E-Surveys (CHERRIES). J Med Internet Res. 2004 Sep 29;6(3):e34 [erratum in J Med Internet Res. 2012; 14(1): e8.]. Article available at https://www.jmir.org/2004/3/e34/; erratum available https://www.jmir.org/2012/1/e8/. Copyright ©Gunther Eysenbach. Originally published in the Journal of Medical Internet Research, 29.9.2004 and 04.01.2012.

Confidential: For Review Only

This is an open-access article distributed under the terms of the Creative Commons Attribution License (https://creativecommons.org/licenses/by/2.0/), which permits unrestricted use, distribution, and reproduction in any medium, provided the original work, first published in the Journal of Medical Internet Research, is properly cited.

Confidential: For Review Only

Supplementary Material Table 1: Parent carers' and school and social care staff's perspectives on how easy specified toileting methods are to use at home, and how easy it is for them to provide or use specified methods to help children and young people with toileting, respectively

**Survey respondents / clinical group**	**Method, n (% of respondents)**	**How easy it is to use the method**									
		**Very easy**		**Easy**		**Difficult**		**Very difficult**		**Never used**	
**Parent carers / non-spinal cord pathology**	Dietary advice (N = 476) *(N = 244)*	37	(8) *(15)*	104	(22) *(43)*	64	(13) *(26)*	39	(8) *(16)*	232	(49)
	Fluid intake advice (N = 491) *(N = 324)*	51	(10) *(16)*	140	(29) *(43)*	101	(21) *(31)*	32	(7) *(10)*	167	(34)
	Behavioural intervention e.g., reward chart, timer, alarm (N = 483) *(N = 292)*	18	(4) *(6)*	72	(15) *(25)*	99	(20) *(34)*	103	(21) *(35)*	191	(40)
	Simple aid/equipment e.g., raised step or seat (N = 479) *(N = 292)*	60	(13) *(21)*	156	(33) *(53)*	54	(11) *(18)*	22	(5) *(8)*	187	(39)
	Bespoke aid/equipment e.g., hoist or frame (N = 456) *(N = 77)*	12	(3) *(16)*	31	(7) *(40)*	22	(5) *(29)*	12	(3) *(16)*	379	(83)
	Housing adaptions e.g., specialised toilet (N = 456) *(N = 68)*	16	(4) *(24)*	23	(5) *(34)*	15	(3) *(22)*	14	(3) *(21)*	388	(85)
	Continence products e.g., nappies, pads, pull ups (N = 497) *(N = 401)*	132	(27) *(33)*	188	(38) *(47)*	60	(12) *(15)*	21	(4) *(5)*	96	(19)
	Medication e.g., laxatives, anticholinergics, desmopressin (N = 489) *(N = 295)*	55	(11) *(19)*	131	(27) *(44)*	73	(15) *(25)*	36	(7) *(12)*	194	(40)
	Catheters (N = 442) *(N = 14)*	2	(<1) *(14)*	3	(<1) *(21)*	5	(1) *(36)*	4	(1) *(29)*	428	(97)
	Colonic enema (bowel washout) (N = 450) *(N = 29)*	4	(1) *(14)*	11	(2) *(38)*	3	(<1) *(10)*	11	(2) *(38)*	421	(94)

Confidential: For Review Only

	Surgical intervention e.g., botox, bladder reconstruction, mitrofanoff (N = 447) *(N = 19)*	1	(<1) *(5)*	6	(1) *(32)*	4	(1) *(21)*	8	(2) *(42)*	428	(96)

		**Very easy**		**Easy**		**Difficult**		**Very difficult**		**Never used**	
**Parent carers / spinal cord pathology**	Dietary advice (N = 18) *(N = 11)*	5	(28) *(45)*	2	(11) *(18)*	4	(22) *(36)*	0	(0) *(0)*	7	(39)
	Fluid intake advice (N = 18) *(N = 13)*	4	(22) *(31)*	3	(17) *(23)*	5	(28) *(38)*	1	(6) *(8)*	5	(28)
	Behavioural intervention e.g., reward chart, timer, alarm (N = 17) *(N = 7)*	0	(0) *(0)*	3	(18) *(43)*	2	(12) *(29)*	2	(12) *(29)*	10	(59)
	Simple aid/equipment e.g., raised step or seat (N = 20) *(N = 12)*	5	(25) *(42)*	3	(15) *(25)*	2	(10) *(17)*	2	(10) *(17)*	8	(40)
	Bespoke aid/equipment e.g., hoist or frame (N = 18) *(N = 4)*	1	(6) *(25)*	1	(6) *(25)*	2	(11) *(50)*	0	(0) *(0)*	14	(78)
	Housing adaptions e.g., specialised toilet (N = 19) *(N = 5)*	2	(11) *(40)*	3	(16) *(60)*	0	(0) *(0)*	0	(0) *(0)*	14	(74)
	Continence products e.g., nappies, pads, pull ups (N = 19) *(N = 16)*	7	(37) *(44)*	8	(42) *(50)*	1	(5) *(6)*	0	(0) *(0)*	3	(16)
	Medication e.g., laxatives, anticholinergics, desmopressin (N = 18) *(N = 17)*	6	(33) *(35)*	4	(22) *(24)*	4	(22) *(24)*	3	(17) *(18)*	1	(6)
	Catheters (N = 19) *(N = 15)*	4	(21) *(27)*	8	(42) *(53)*	2	(11) *(13)*	1	(5) *(7)*	4	(21)
	Colonic enema (bowel washout) (N = 18) *(N = 11)*	1	(6) *(9)*	6	(33) *(55)*	1	(6) *(9)*	3	(17) *(27)*	7	(39)
	Surgical intervention e.g., botox, bladder reconstruction, mitrofanoff (N = 18) *(N = 6)*	2	(11) *(33)*	2	(11) *(33)*	1	(6) *(17)*	1	(6) *(17)*	12	(67)

Confidential: For Review Only

		**Very easy**		**Easy**		**Difficult**		**Very difficult**		**Never used**	
**School and social care staff / not separated by clinical group**	Dietary advice (N = 116) *(N = 96)*	17	(15) *(18)*	59	(51) *(61)*	19	(16) *(20)*	1	(1) *(1)*	20	(17)
	Fluid intake advice (N = 116) *(N = 106)*	24	(21) *(23)*	64	(55) *(60)*	16	(14) *(15)*	2	(2) *(2)*	10	(9)
	Behavioural intervention e.g., reward charts, timers, alarms (N = 115) *(N = 109)*	46	(40) *(42)*	52	(45) *(48)*	10	(9) *(9)*	1	(1) *(1)*	6	(5)
	Physical aids / equipment e.g., specialised toilets, hoists, frames (N = 116) *(N = 99)*	30	(26) *(30)*	47	(41) *(47)*	15	(13) *(15)*	7	(6) *(7)*	17	(15)
	Continence products e.g., nappies, pads, pull ups, continence pants (N = 114) *(N = 102)*	34	(30) *(33)*	49	(43) *(48)*	15	(13) *(15)*	4	(4) *(4)*	12	(11)
	Medication or drugs e.g., laxatives, anticholinergics, desmopressin (N = 113) *(N = 72)*	15	(13) *(21)*	37	(33) *(51)*	19	(17) *(26)*	1	(1) *(1)*	41	(36)
	Catheterisation (N = 113) *(N = 31)*	8	(7) *(26)*	11	(10) *(35)*	9	(8) *(29)*	3	(3) *(10)*	82	(73)
	Colonic enema (bowel washout) (N = 112) *(N = 21)*	1	(1) *(5)*	5	(4) *(24)*	12	(11) *(57)*	3	(3) *(14)*	91	(81)
	Surgical intervention e.g., cytoscopic botox, ACE, bladder reconstruction, mitrofanoff (N = 109) *(N = 16)*	2	(2) *(13)*	4	(4) *(25)*	8	(7) *(50)*	2	(2) *(13)*	93	(85)

Numbers in italic show the total number of respondents (*N*) and percentage of respondents choosing the response option, excluding those who answered 'Never used'. ACE = antegrade colonic enema.

Confidential: For Review Only

Supplementary Material Table 2: Health professionals', school and social care staff's, and parent carers' perspectives on provision of interventions to families in their area

Response option, n (% of respondent group)	Health professionals												School and social care staff		Parent carers			
	BBS Nurses		Nurses		Paediatricians		Surgeons		Therapists		Other				Non-spinal cord pathology		Spinal cord pathology	
**Behavioural - e.g., timer, alarm**	**N = 20**		**N = 74**		**N = 29**		**N = 15**		**N = 27**		**N = 12**		**N = 112**		**N = 519**		**N = 17**	
Free of charge	12	(60)	26	(35)	14	(48)	5	(33)	5	(19)	4	(33)	22	(20)	29	(6)	0	(0)
Subsidised	0	(0)	0	(0)	2	(7)	0	(0)	1	(4)	1	(8)	3	(3)	1	(<1)	0	(0)
Available to purchase	0	(0)	10	(14)	0	(0)	1	(7)	4	(15)	0	(0)	18	(16)	46	(9)	0	(0)
Unavailable	6	(30)	26	(35)	4	(14)	6	(40)	11	(41)	6	(50)	14	(13)	49	(9)	2	(12)
Don't know (/ never used)**	2	(10)	12	(16)	9	(31)	3	(20)	6	(22)	1	(8)	55	(49)	394	(76)	15	(88)
**Simple aid/equipment - e.g., raised seat or step**	**N = 18**		**N = 74**		**N = 29**		**N = 15**		**N = 27**		**N = 12**		**N = 113***		**N = 523**		**N = 19**	
Free of charge	7	(39)	27	(36)	11	(38)	2	(13)	20	(74)	3	(25)	43	(38)	91	(17)	8	(42)
Subsidised	1	(6)	1	(1)	0	(0)	1	(7)	0	(0)	0	(0)	7	(6)	2	(<1)	0	(0)
Available to purchase	2	(11)	12	(16)	7	(24)	1	(7)	1	(4)	1	(8)	18	(16)	69	(13)	2	(11)
Unavailable	5	(28)	25	(34)	3	(10)	6	(40)	3	(11)	5	(42)	13	(12)	45	(9)	2	(11)
Don't know (/ never used)**	3	(17)	9	(12)	8	(28)	5	(33)	3	(11)	3	(25)	32	(28)	316	(60)	7	(37)
**Bespoke aid/equipment - e.g., hoist or frame**	**N = 18**		**N = 74**		**N = 29**		**N = 15**		**N = 27**		**N = 12**				**N = 521**		**N = 17**	
Free of charge	9	(50)	42	(57)	18	(62)	0	(0)	20	(74)	2	(17)			73	(14)	2	(12)

Confidential: For Review Only

Subsidised	2	(11)	2	(3)	0	(0)	1	(7)	1	(4)	1	(8)			5	(1)	0	(0)
Available to purchase	1	(6)	3	(4)	0	(0)	0	(0)	2	(7)	0	(0)			12	(2)	0	(0)
Unavailable	3	(17)	15	(20)	1	(3)	6	(40)	1	(4)	4	(33)			20	(4)	0	(0)
Don't know (/ never used)**	3	(17)	12	(16)	10	(34)	8	(53)	3	(11)	5	(42)			411	(79)	15	(88)
**Housing adaption - e.g., specialised toilet**	**N = 18**		**N = 72**		**N = 29**		**N = 15**		**N = 27**		**N = 12**		**N = 113**		**N = 516**		**N = 19**	
Free of charge	7	(39)	26	(36)	15	(52)	0	(0)	13	(48)	3	(25)	29	(26)	61	(12)	5	(26)
Subsidised	3	(17)	5	(7)	3	(10)	1	(7)	4	(15)	1	(8)	14	(12)	19	(4)	1	(5)
Available to purchase	1	(6)	4	(6)	0	(0)	0	(0)	2	(7)	0	(0)	9	(8)	14	(3)	1	(5)
Unavailable	3	(17)	14	(19)	1	(3)	6	(40)	4	(15)	4	(33)	11	(10)	20	(4)	0	(0)
Don't know (/ never used)**	4	(22)	23	(32)	10	(34)	8	(53)	4	(15)	4	(33)	50	(44)	402	(78)	12	(63)
**Products - e.g., pads, nappies, continence pants, pull ups**	**N = 18**		**N = 72**		**N = 29**		**N = 14**		**N = 26**		**N = 12**		**N = 115**		**N = 534**		**N = 19**	
Free of charge	17	(94)	53	(74)	19	(66)	8	(57)	11	(42)	6	(50)	55	(48)	256	(48)	9	(47)
Subsidised	1	(6)	5	(7)	4	(14)	0	(0)	4	(15)	1	(8)	8	(7)	21	(4)	1	(5)
Available to purchase	0	(0)	2	(3)	1	(3)	0	(0)	0	(0)	0	(0)	17	(15)	55	(10)	6	(32)
Unavailable	0	(0)	7	(10)	0	(0)	3	(21)	2	(8)	4	(33)	13	(11)	42	(8)	1	(5)
Don't know (/ never used)**	0	(0)	5	(7)	5	(17)	3	(21)	9	(35)	1	(8)	22	(19)	160	(30)	2	(11)
**Medications - e.g., laxatives**	**N = 18**		**N = 72**		**N = 29**		**N = 14**		**N = 26**		**N = 12**		**N = 113**		**N = 522**		**N = 18**	
Free of charge	15	(83)	60	(83)	29	(100)	13	(93)	8	(31)	9	(75)	47	(42)	290	(56)	17	(94)
Subsidised	1	(6)	0	(0)	0	(0)	1	(7)	0	(0)	0	(0)	2	(2)	3	(<1)	0	(0)
Available to purchase	0	(0)	0	(0)	0	(0)	0	(0)	0	(0)	1	(8)	7	(6)	15	(3)	0	(0)

Confidential: For Review Only

Unavailable	1	(6)	10	(14)	0	(0)	0	(0)	2	(8)	2	(17)	15	(13)	6	(1)	0	(0)
Don't know (/ never used)**	1	(6)	2	(3)	0	(0)	0	(0)	16	(62)	0	(0)	42	(37)	208	(40)	1	(6)

* = Aids/equipment not separated into simple or bespoke in school and social care survey; ** = response option includes *never used* for school and social care staff and parent carer surveys; BBS = Bladder and Bowel Specialist.

Confidential: For Review Only
