# Supplementary material for: Interventions to improve continence for children and young people with neurodisability: a national survey of practitioner and family perspectives and experiences
Source: BMJ Paediatr Open. 2024 Jan 19;8(1):e002238. doi: 10.1136/bmjpo-2023-002238 (PMC10806478; doi:10.1136/bmjpo-2023-002238)
Supplement: Reviewer comments [file bmjpo-2023-002238.reviewer_comments.pdf]

PEER REVIEW HISTORY

BMJ Paediatrics Open publishes all reviews undertaken for accepted manuscripts. Reviewers are asked to complete a checklist review form and are provided with free text boxes to elaborate on their assessment. These free text comments are reproduced below.

ARTICLE DETAILS

**TITLE (PROVISIONAL)**	Heated and Humidified High Flow Therapy (HHHFT) in extreme and very preterm neonates with respiratory distress syndrome (RDS): a retrospective cohort from a tertiary care setting in Pakistan
**AUTHORS**	Kessani, Vikram<br>Hemani, Fatima<br>Ali, Iqrar<br>Niaz, Sana<br>Somroo, Albar<br>Zaheer, Rija<br>Das, Jai<br>Haque, Khalid<br>Ali, Syed

VERSION 1 – REVIEW

**REVIEWER**	Prof. Junaid Muhib<br>Sheikh Shakhbout Medical City, Pdiatrics/NICU
**REVIEW RETURNED**	07-Jul-2023

**GENERAL COMMENTS**	This is a good effort from LMIC country and the results are very encouraging only issue is these babies are relatively mature (32 wks) and their birth wt also above 1500gms (1600+gm)<br>Next, try to do it lower gestation age babies

**REVIEWER**	Dr. Camilla Gizzi<br>Sandro Petrini Hospital, Pediatrics and Neonatology
**REVIEW RETURNED**	16-Jul-2023

**GENERAL COMMENTS**	In this study, the authors report on their experience on HHHFNC in a single tertiary centre in Karaci, Pakistan. Data illustrated are interesting and in my opinion worthy of publication, nevertheless the manuscript needs both major and minor revisions.<br>My comments are:<br>Overall, the manuscript would benefit of an English language editing and also references should be checked carefully by the authors, as there are many imprecisions.<br>Abstract:<br>Pag.4

Objectives - Line 9: I suggest to modify as "….in spontaneously breathing moderate-late, very, and extremely preterm infants……" as the cohort included infants with GA >32 weeks
Patients - Line 20: I suggest to modify as "lung diseases other than RDS" among exclusion criteria
Main outcome measures - Line 34: I suggest to add the word "Effectiveness" at the beginning of the sentence and again "in moderate-late, very, and extremely preterm infants". Line 36: why the authors write "and comparison with moderate-late, preterm neonates"? Where the comparison is made within the manuscript?
Results: please give a comment on the results also on comparisons of the results between very-extremely preterm with late-moderate preterm neonates
From line 53 should be Conclusions - I suggest to follow an order for the grade of prematurity as used previously, that is: late, moderate-late, very, and extremely preterm infants

Introduction:
Pag. 6 Line 16: please chose between "is" and "remains" - Lines 18 and 23: please, verify if references 4 and 5 are correctly cited within the context - Lines 43-52: classification should include also NIPPV, that is non-synchronized nasal intermittent positive pressure ventilation
Pag. 7 Line 40: what the authors mean by "gestation outcomes?", please give some examples – Line 42: "in preterm neonates" is pleonastic as this have been previously written.

Methodology:
Pag. 8 Sample size: please give more details on the rationale for choosing the sample size. The authors compare nHFT with NCPAP but also with invasive ventilation. For which category of infants? And how is defined non-invasive and invasive ventilation failure? According to which data, literature data or authors' unit data?

Study setting:
Page 9 Line 3: please give more details on "prophylactic caffeine", do the authors administer caffeine in the delivery room or in the unit? If in the unit, within how many hours of life? - Line 29 and 31: please type PCO2 correctly. Line 33: SpO2 target is >95% for all GA infants?
This section would benefit from an English language editing.
Page 10 Line 39-48: among exclusion criteria, please see my comment given for the abstract section – Line 39: please explain better this sentence, what does patient public involvement mean?

Results:
Authors can shorten the results section jut referring to the Tab. 1 and describing only the most relevant information. In this section author should mention and refer also to Table 2, describing the more relevant results.

	Discussion:<br>Page 13 Lines 6-8: “Our study”, instead of “A study”. Again, where in the manuscript the comparison between very-extremely pretem infants and moderately-late preterm infants is reported? – Line 33: reference 18 in not correct, please add the correct one – Lines 37-39: “HHHFT has not been used as primary respiratory support in preterm neonates less than 32 weeks of gestation” This sentence is incorrect, please verify the GA of treated infants in references 19 and 20.<br>Page 14 Lines 2-22: This period should be better rewritten. Moreover, comments on the incidence of BPD should be limited to the very-extreme preterm infants, excluding the late-moderate ones.<br><br>Conclusions:<br>Page 15 Line 10: “HHHFNC can be used also in extreme and very preterm infants…….”<br><br>Page 16 Lines 24-27: What his study adds? It is not clear, less than what?<br><br>References:<br>Page 17-18: check carefully all the given references<br><br>Tables:<br>Pag 19<br>Tab. 1 Authors can shorten the results section jut referring to the Tab. 1 and describing only the most relevant information. Within the table 1, I would eliminate the term “incredibly” and live only “extremely low birth weight”. Among “Data distribution of independent variables”, please give all the results as median (range), moreover specify FiO2 referred to when (i.e beginning of HHHFNC, surfactant administration, or? In the Figure legend give a reference for the radiological grading of RDS. Why the authors write Tab. 2 within the Tab.1? Please modify.<br>Pag 20<br>Tab. 2 please give the statistical significance of the reported results, between >32 and <32 weeks. Moreover the classification should be: equal to or above 32 wees and below 32 weeks

VERSION 1 – AUTHOR RESPONSE

Comments of reviewer number 1.

- The reviewer’s comment that the babies in our cohort were relatively mature i.e., more than 32-week gestation. In fact, as shown in Table 1 there were 58.7% babies below the gestation of 32 weeks in our cohort

Comments of reviewer number 2.

- We have tried our best to improve English language of our manuscript.
- Objectives, Line 9 has been modified according to the reviewer's suggestions; "in spontaneously breathing moderate-late, very and extremely preterm infants"
- Patients, Line 20 has been modified as "lung disease other than RDS" among exclusion criteria
- Main outcome measures, Line 34 the word effectiveness has been added at the beginning of the sentence and added "in moderate-late, very and extremely preterm infants"
- Line 36: Has been removed
- Results: We have given comparative results between less than and more than 32 weeks gestation infants in Table 3
- Introduction; Page 6 Line 16. Corrected as suggested by the reviewer
- Lines 18 and 23. References have been checked, a new reference added and their correct citation has been checked
- Lines 43-52: Classification now includes NIPPV as non-synchronised nasal intermittent positive pressure ventilation
- Page 7 Lines 40 and 42; amended as per reviewer's comments as we understood them
- Methodology : Details about sample size have been provided
- Study setting: Details about caffeine therapy have been provided
- We have corrected PCO2 and SpO2 to PCO_2 , SpO_2
- Page 10, Line 39-48 corrected according to reviewer's suggestions
- Line 39. Patient-public involvement has been deleted
- Results: We have shortened the results section and refer to tables
- Discussion: Page 13, lines 6-8. Corrected
- Line 33. Reference has been corrected
- Lines 37-39; statement has been corrected
- Page 14, Lines 2-22. We have rephrased this part and included statement er BPD
- Conclusion: Page 15 Line 10: Corrected
- References: Rechecked and verified
- Tables: We have modified the tables according to the reviewer's advice as we understood them.

I hope this meets the requirements stated in your mail and the reviewer's comments.

Yours truly,

Dr. V Kessani

VERSION 2 – REVIEW

**REVIEWER**	Prof. Junaid Muhib<br>Sheikh Shakhbout Medical City, Pdiatrics/NICU
**REVIEW RETURNED**	27-Sep-2023

**GENERAL COMMENTS**	The study is very well done but still, we cannot conclude that HHFNC can be used as primary support for <28 weeks of babies.

	Statistically, it showed significance as a larger number around 80% of the population is above 30 weeks.

VERSION 2 – AUTHOR RESPONSE

NONE
